# Physiologically based kinetic modelling based prediction of in vivo rat and human acetylcholinesterase (AChE) inhibition upon exposure to diazinon

**DOI:** 10.1007/s00204-021-03015-1

**Published:** 2021-03-14

**Authors:** Shensheng Zhao, Sebastiaan Wesseling, Bert Spenkelink, Ivonne M. C. M. Rietjens

**Affiliations:** Division of Toxicology, Wageningen University and Research, Stippeneng 4, 6708 WE Wageningen, The Netherlands

**Keywords:** Toxic equivalency factor (TEF), Acetylcholinesterase (AChE) inhibition, Diazinon (DZN), Physiologically based kinetic (PBK) modelling, Reverse dosimetry, Quantitative in vitro to in vivo extrapolation (QIVIVE)

## Abstract

The present study predicts in vivo human and rat red blood cell (RBC) acetylcholinesterase (AChE) inhibition upon diazinon (DZN) exposure using physiological based kinetic (PBK) modelling-facilitated reverse dosimetry. Due to the fact that both DZN and its oxon metabolite diazoxon (DZO) can inhibit AChE, a toxic equivalency factor (TEF) was included in the PBK model to combine the effect of DZN and DZO when predicting in vivo AChE inhibition. The PBK models were defined based on kinetic constants derived from in vitro incubations with liver fractions or plasma of rat and human, and were used to translate in vitro concentration–response curves for AChE inhibition obtained in the current study to predicted in vivo dose–response curves. The predicted dose–response curves for rat matched available in vivo data on AChE inhibition, and the benchmark dose lower confidence limits for 10% inhibition (BMDL_10_ values) were in line with the reported BMDL_10_ values. Humans were predicted to be 6-fold more sensitive than rats in terms of AChE inhibition, mainly because of inter-species differences in toxicokinetics. It is concluded that the TEF-coded DZN PBK model combined with quantitative in vitro to in vivo extrapolation (QIVIVE) provides an adequate approach to predict RBC AChE inhibition upon acute oral DZN exposure, and can provide an alternative testing strategy for derivation of a point of departure (POD) in risk assessment.

**Supplementary Information:**

The online version contains supplementary material available at 10.1007/s00204-021-03015-1.

## Introduction

**Fig. 1:**
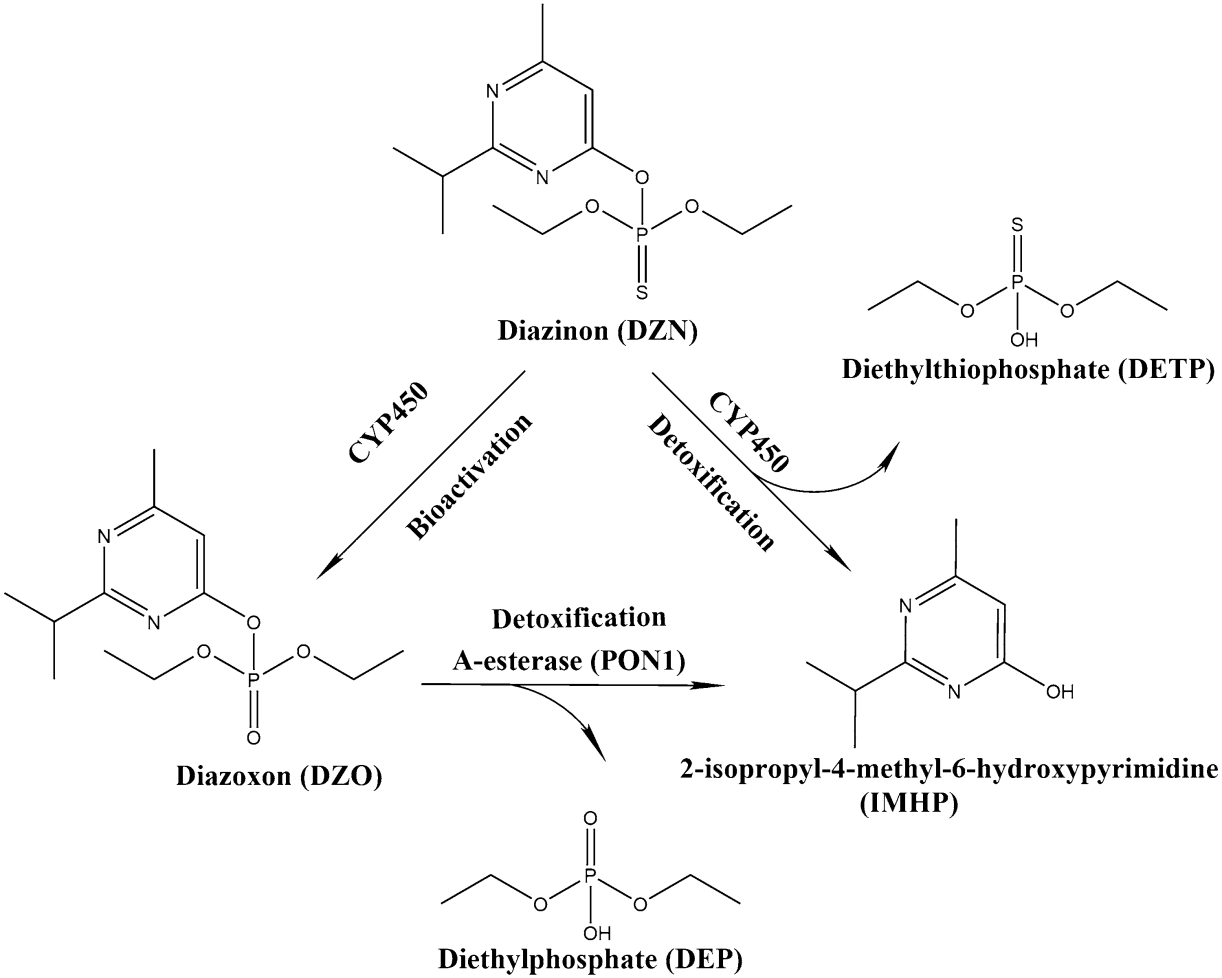
Biotransformation pathways of DZN. PON1, Paraoxonase 1; CYP450, cytochrome P450

Diazinon (DZN) is the common name for *O,O*-diethyl *O*-2-isopropyl-6-methylpyrimidin-4-yl phosphorothioate (Fig. 1), which is used as a pesticide in agriculture or veterinary medicine (JMPR 2016). DZN belongs to the class of thiophosphate insecticides for which acute toxicity is associated with irreversible inhibition of acetylcholinesterase (AChE) resulting in accumulation of acetylcholine at the synaptic cleft (Colovic et al. 2013). Different physiological symptoms such as headache, abdominal cramps, difficulty in breathing, and even death can result from acute DZN exposure (Burgess et al. 2008). Beside AChE, other B-esterases such as butyrylcholinesterase (BuChE) and carboxylesterase (CaE) can also be inhibited by DZN exposure (Poet et al. 2004). Although little is known about the profile of BuChE and CaE in human, it is known that inhibition of BuChE does not induce toxic effects (Jokanović 2009; Jokanović et al. 2020).

Upon oral administration, DZN undergoes multiple metabolic pathways (Fig. 1) in different tissues, particularly in the liver, due to the high abundancy of cytochromes P450 (CYP450) in this organ (Ellison et al. 2012; Sams et al. 2004). Previous studies reported that CYP450 are capable of bioactivating DZN to its active oxon metabolite diazoxon (DZO) (Fig. 1) which is a stronger AChE inhibitor than DZN, and of detoxifying DZN to 2-isopropyl-4-methyl-6-hydroxypyrimidine (IMHP) and diethylthiophosphate (DETP) (Fig. 1) (Ellison et al. 2012; Mutch and Williams 2006; Sams et al. 2004). In human, the bioactivation of DZN is mediated by especially CYP2B6, CYP2C19 and CYP3A4, while the detoxification is catalysed by CYP1A1, CYP1A2, CYP2B6, CYP2C19 and CYP3A4 (Ellison et al. 2012; Kappers et al. 2001; Mutch and Williams 2006; Sams et al. 2004). Paraoxonase 1 (PON1) is another enzyme involved in biotransformation of DZN, catalysing detoxification of DZO to IMHP and diethylphosphate (DEP) (Fig. 1). Different from CYP450-mediated conversions, PON1-mediated detoxification occurs not mainly in the liver but also in blood due to the excretion of PON1 from liver to blood (Pyati et al. 2015).

To date, the point of departure (POD) to define an acute reference dose (ARfD) for risk assessment of acute exposure to DZN is based on the no-observed-adverse-effect level (NOAEL) of acute AChE inhibition and neurotoxicity in rats (EFSA 2006; JMPR 2016), or on the BMDL_10_, the lower confidence limit of the benchmark dose (BMD) causing 10% inhibition of red blood cell (RBC) AChE activity in in vivo animal experiments (USEPA 2016), because of the absence of adequate human data. However, the use of animal data to define health based guidance values may not (fully) reflect the human situation (Martignoni et al. 2006). To overcome this issue, alternative testing strategies can be considered, including physiologically based kinetic (PBK) modelling-facilitated reverse dosimetry (Louisse et al. 2017) that enables quantitative in vitro to in vivo extrapolation (QIVIVE), as a potential novel approach in risk assessment. The PBK modelling-based alternative approach has been successfully used to predict chlorpyrifos-related AChE inhibition (Timchalk et al. 2002; Zhao et al. 2019) and also a variety of other chemical-induced adverse effects including for example cardiotoxicity induced by methadone, liver toxicity induced by pyrrolizidine alkaloids and developmental toxicity of retinoids, glycolethers and phenols (Boonpawa et al. 2017; Louisse et al. 2010; Ning et al. 2019; Shi et al. 2020; Strikwold et al. 2013, 2017). In case of DZN, previously a physiologically based pharmacokinetic and pharmacodynamic model was developed for both human and rat (Poet et al. 2004). However, in this previous study, the predicted AChE inhibition was assumed to be caused by the metabolite DZO only, not taking the contribution of the parent compound DZN on AChE inhibition into account. Furthermore, the kinetic parameters were solely determined using rat liver microsomes and scaled for further use in rat and human PBK models, while the kinetic parameters for DZO detoxification in blood were assumed to be equal to those in liver. Apart from that, the model was not used to define an in vivo dose response curve for AChE inhibition from which a POD for human risk assessment could be derived.

Therefore, the aim of the present study was to assess the possibility of using mainly in vitro and in-silico data as input for PBK modelling facilitated QIVIVE to derive a POD for acute toxicity of DZN. The kinetic parameters of DZN biotransformation for rat and human PBK models were determined in a species-specific way by incubating a range of concentrations of DZN or DZO with rat or human liver microsomes and plasma. Considering that both DZN and DZO are able to inhibit AChE, a toxic equivalency factor (TEF) approach was employed and incorporated into the PBK model to describe the internal combined effective concentration of DZN and DZO in DZO equivalents. The TEF-coded PBK model was subsequently used to translate in vitro concentration–response curves for DZO concentration-dependent inhibition of rat AChE or recombinant human AChE (rhAChE) to predicted in-vivo dose–response curves for DZN exposure mediated RBC AChE inhibition enabling definition of a BMDL_10_ as POD for risk assessment and quantification of potential inter-species differences between rat and human.

## Materials and methods

### Materials

#### Chemicals

Diazinon (DZN), 2-isopropyl-4-methyl-6-hydroxypyrimidine (IMHP), acetylthiocholine iodide (ATC), 5,5′-dithiobis (2-nitrobenzoic acid) (DTNB), bovine serum albumin (BSA), tetraisopropyl pyrophosphoramide (iso-OMPA), reduced nicotinamide adenine dinucleotide phosphate (NADPH) and Trizma^®^base were purchased from Sigma-Aldrich (Zwijndrecht, Switzerland). Diazoxon (DZO) was purchased from TRC-Canada (Toronto, Ontario, Canada). Magnesium chloride hexahydrate (MgCl_2_·6H_2_O), hydrochloric acid (HCl), sodium hydroxide (NaOH), ethylenediaminetetraacetic acid disodium salt dihydrate (EDTA) and calcium chloride dihydrate (CaCl_2_·2H_2_O) were purchased from VWR International (Amsterdam, The Netherlands). Acetonitrile (ACN, UPLC/MS grade) and methanol (UPLC/MS grade) were purchased from Biosolve (Valkenswaard, The Netherlands). Rapid equilibrium dialysis (RED) materials (RED inserts, RED based plate and sealing tape), and Pierce™ BCA protein assay kit were purchased from Thermo Fisher Scientific (Rockford, IL, USA). Phosphate-buffered saline (PBS pH 7.4 (1X)) was purchased from GIBCO (Paisley, UK).

#### Biological material

Human liver microsomes (pooled from 20 donors, mixed gender) and rat liver microsomes (Sprague–Dawley, male) were purchased from Corning (Amsterdam, The Netherlands). Human plasma (pooled from 6 donors, mixed gender) were purchased from Zen-Bio, Inc (NC, USA). Rat plasma (Sprague–Dawley) was purchased from Innovative Research Inc. (MI, USA). For the rat samples further information on number of animals used to create the samples (liver microsomes and plasma) or on gender (plasma) was not provided by the provider. Rat blood was purchased from BioIVT (West Sussex, UK). Recombinant human AChE was purchased from Sigma-Aldrich Co. (St. Louis, MO, USA), and the enzyme was stabilized with 1 mg/ml BSA.

### Methods

#### Protein determination

The total protein concentration of human and rat plasma was measured using a Pierce™ BCA protein assay kit (ThermoFisher Scientific 2020). The experiment was conducted using the manufacturer’s protocol. In detail, 25 µl sample or protein standard solution were incubated with 200 µl working reagents in a 96-well plate at 37 ℃ for 30 min. Next, the plate was cooled to room temperature, followed by measuring the absorbance at 562 nm for each sample or protein standard. The protein concentration of the unknown sample was quantified based on the calibration curve (protein concentration versus 562 nm absorbance value) generated with the protein standards.

### In vitro metabolic incubations for deriving kinetic parameters

The in vitro incubations for investigating CYP450-mediated biotransformation of DZN were performed using rat and human liver microsomes based on the method described by Sams et al. (2004) and Smith et al. (2011) with some modifications. Preliminary studies were carried out to optimize both incubation time and microsomal protein concentration, to define conditions at which metabolism was linear with respect to time and the amount of microsomal protein (data not shown) to be used for further kinetic studies. The final incubations contained 50 mM Tris–HCl (pH 7.4), 5 mM MgCl_2_, 1 mM EDTA (as an A-esterase PON1 inhibitor) (Bizoń and Milnerowicz 2018), 50 µM iso-OMPA (as a B-esterases inhibitor) (Lane et al. 2006), 1 mM NADPH, and DZN at final concentrations of 1, 2.5, 5, 10, 25, 50, 100 and 250 µM (added from 100 times concentrated stock solutions in methanol). In these incubations, the formed DZO might also be further hydrolyzed to IMHP by esterases present in the liver microsomes, hampering accurate quantification of the formation of DZO or IMHP by the CYP450-mediated reactions. Therefore, to adequately define the CYP450-mediated conversion from DZN to DZO and from DZN to IMHP, esterase inhibitors (EDTA and iso-OMPA) were added to prevent this ‘untargeted’ conversion of DZO to IMHP in these microsomal incubations. Addition of esterase inhibitors EDTA and iso-OMPA has been commonly applied when studying CYP450-mediated conversion of organophosphate (OP) pesticides in liver microsomal incubations for determining kinetic parameters for their CYP450-mediated pathways (Buratti et al. 2005; Dadson et al. 2013; Ellison et al. 2012; Foxenberg et al. 2007; Poet et al. 2003; Smith et al. 2011). Based on previous studies, CYP450-mediated activities are not adversely affected by the addition of these inhibitors (1 mM EDTA and 50 µM iso-OMPA) to the microsomal incubations (Buratti et al. 2003; Rasmussen 2012). After 1 min preincubation in a 37 °C water bath, 2.5 µl of human or rat liver microsomes (final concentration 0.25 mg microsomal protein/ml) were added to initiate the reaction. The total incubation mixture was 200 µl. Control incubations were carried out by replacing NADPH with buffer. The reaction was terminated after 2.5 min by adding 200 µl ice-cold ACN. After the incubation, samples were centrifuged at 16000 g (4℃) for 5 min, and supernatants of rat samples were further diluted 2 × in a mixture of ACN and 50 mM Tris–HCl (pH 7.4) (ratio 1:1, v/v). At the end, both diluted rat sample supernatants and undiluted human sample supernatants were analysed by LC–MS/MS for quantification of metabolite formation.

The in vitro incubations for quantification of kinetic parameters for PON1-mediated detoxification of DZO were conducted using both liver microsomes and plasma from either human or rat based on the method from Poet et al. (2003) with some modifications. It should be noted that the enzyme activity detected in these DZO detoxification incubations with tissue fractions could in theory be due to various enzymes, but based on literature data (Costa et al. 1999; Jokanović et al. 2020; Poet et al. 2004), the activity can be mainly ascribed to the activity of PON1. Preliminary studies were carried to optimize and select the incubation conditions with respect to linearity for both incubation time and microsomal/plasma protein concentration (data not shown). The final incubations contained 2 mM CaCl_2_ in 50 mM Tris–HCl (pH 7.4) and DZO at final concentrations of 0.5, 1, 2.5, 5, 10, 25, 50, 100, 250, 500 and 1000 µM (added from 100 times concentrated stock solutions in methanol). After 1 min preincubation in a 37 °C water bath, 1 µl of human or rat liver microsomes (final concentration 0.1 mg microsomal protein/ml), or 1 µl of human or rat plasma (final concentration 0.385 mg plasma protein/ml for human plasma and 0.300 mg plasma protein/ml for rat plasma) was added to initiate the reaction. The total incubation mixture was 200 µl. Control incubations were carried out by replacing liver microsomes or plasma with buffer. The reaction was terminated after 1 min incubation (human samples) or 2 min incubation (rat samples) by adding 200 µl ice-cold ACN. Subsequently, samples were centrifuged at 16000 g (4℃) for 5 min, and supernatants were further diluted (30 × for liver sample and 40 × for plasma sample) in a mixture of ACN and 50 mM Tris–HCl (pH 7.4) (ratio 1:1, v/v) before analysis by LC–MS/MS for quantification of metabolite formation.

### Quantification of DZN and DZO metabolites by LC–MS/MS

The amounts of parent compound DZN and formed DZO and IMHP in samples from the microsomal and plasma incubations were identified and quantified using a Shimadzu Nexera XR LC-20AD-xr UHPLC system coupled to a Shimadzu LCMS-8045 mass spectrometer (Kyoto, Japan) equipped with an electrospray ionization (ESI) interface. The chromatographic separations were conducted on a Kinetex^®^ 1.7 µm Phenyl-Hexyl 100 Å LC column (100 × 2.1 mm). The injection volume was 1 µl at a flow rate of 0.3 ml/min. The temperature of the column was kept at 40℃. The mobile phase A consisted of ultrapure water with 0.1% (v/v) formic acid, and mobile phase B consisted of acetonitrile (ACN) with 0.1% (v/v) formic acid. The gradient started with 0% B and was linearly increased to 100% B in 12 min, kept at 100% B for 1 min and then changed to the initial condition (0% B) at 13.5 min and kept for 5.5 min to re-equilibrate the column before the next injection. The instrument was used in positive mode with multiple reaction monitoring (MRM). The optimized acquisition parameters for DZN, DZO and the metabolite IMHP are listed in Supplementary data I.

### Calculation of kinetic parameters

The Michaelis–Menten parameters for conversion of DZN to DZO and of DZN to IMHP in incubations with liver microsomes, and of DZO to IMHP in incubations with liver or plasma samples were determined by fitting the data to Eq. (1):

1 $$ v = \frac{{V_{\max } \times \left[ S \right] }}{{\left( {K_{{\text{m}}} + \left[ S \right]} \right) }}, $$

where *v* represents the rate of reaction in nmol/min/mg microsomal protein or in nmol/min/mg plasma protein, *S* represents the substrate concentration (in µM), *K*_m_ the apparent Michaelis–Menten constant in µM, and *V*_max_ the apparent maximum rate in nmol/min/mg microsomal protein or in nmol/min/mg plasma protein. The calculation was done using GraphPad Prism 5 for Windows, version 5.04 (GraphPad software, San Diego California USA).

### In vitro AChE inhibition assay to derive concentration–response curves

#### Preparation of rat RBC AChE

Rat RBC AChE was prepared according to the protocol previously described (Dodge et al. 1963; Patel et al. 2000) with some modifications. In the current study, 7 ml rat whole blood was first centrifuged at 2000 g for 10 min (4 °C) to separate plasma and RBCs (pellet). Subsequently, the RBCs were suspended in 5 ml 0.9% saline (sodium chloride) and centrifuged at 2000*g* for 10 min (4 °C) to wash away plasma residue. After three of these washing steps, the washed RBCs were resuspended in 2 ml PBS and lysed by addition of 18 ml lysis buffer (20 mM sodium phosphate, pH 7.4) and freezing at – 80 °C for 24 h. The lysed RBCs were defrozen and the membrane fraction (containing AChE) was sedimented by centrifugation at 20,000*g* for 40 min, and the supernatant was carefully removed. The pellet was washed for another two times by resuspending in lysis buffer and sedimenting as described above. Afterwards the pellet was resuspended in 1 ml 100 mM sodium phosphate (pH 7.4) and was successively centrifuged for 2 min at 2000 g using a Microcentrifuge (VWR, Mini start silverline) to obtain the AChE (supernatant). The enzyme concentration of the isolated rat AChE, expressed in mU/ml, was quantified based on the calibration curve generated using the commercially available rhAChE.

#### AChE activity assay

In the present study, AChE activity of rhAChE and of the extracted rat RBC AChE were used to characterize the inhibitory potency of DZO or DZN on human and rat RBC AChE, based on the protocol from Ellman et al. (1961). Recombinant human AChE was used since it is easy to use (no lysing and washing steps are required compared to use of AChE extracted from native red blood cell), the obtained results will not be affected by haemoglobin (George and Abernethy 1983), and its characteristics are comparable with those of human natural RBC AChE in term of sensitivity towards OP (Amitai et al. 1998; Velan et al. 1991). However, since recombinant rat AChE was not commercially available, extracted rat RBC AChE was used. Preliminary studies were carried out to select the rhAChE enzyme concentration and substrate acetylthiocholine iodide (ATC) concentration that would be within the linear range with respect to formation of the yellow-coloured product 5-thio-2-nitrobenzoic acid (data not shown) and thus optimal to quantify the AChE activity upon inhibition by DZO or DZN. The concentration of DTNB was calculated based on the concentration ratio (ATC/DTNB) of 2 (Stern et al. 2014). To this end, series of increasing concentrations of DZO or DZN in ethanol, 5000 µM chlorpyrifos-oxon in ethanol (CPO, positive control) and 100% ethanol (solvent control) were all diluted 50 × in 100 mM sodium phosphate (pH 7.4) containing 0.1 mg/ml BSA. Incubation was conducted in 96-well plates with 44 µl 100 mM sodium phosphate (pH 7.4) containing 0.1 mg/ml BSA, and 5 µl DZO solution (final concentrations 0.0005, 0.001, 0.005, 0.01, 0.025, 0.05 0.1, 0.25 0.5 and 1 µM), or DZN solution (final concentrations 1, 2.5, 5, 10, 25, 50, 100, 500 and 1000 µM) or 5 µl positive control (CPO at a final concentration of 10 µM) or 5 µl solvent control (ethanol at a final concentration 0.2%). To initiate the inhibition reaction, 1 µl rhAChE or rat AChE (final concentration 0.6 mU/ml) was added to the incubation. The total incubation volume was 50 µl. After 15 min incubation at 37℃, 150 µl reaction reagents (mixture of ATC at a final concentration of 150 µM and DTNB at a final concentration of 75 µM) were added into each well. The final total volume of each well was 200 µl. Subsequently, the 96-well plate was measured continuously for 10 min at absorbance 412 nm at 37℃ to quantify the remaining AChE activity.

#### AChE activity data analysis

The rhAChE and rat AChE activity were expressed as the remaining AChE activity relative to solvent control (100% activity) and positive control (0% activity) based on Eq. (2):

2 $$ {\text{AChE}}\,{\text{activity\% }} = \frac{{A412\left( {t10 - t0} \right){\text{test}}\,{\text{compound}} - A412\left( {t10 - t0} \right){\text{positive}}\,{\text{control}}}}{{A412\left( {t10 - t0} \right){\text{solvent}}\,{\text{control}} - A412\left( {t10 - t0} \right){\text{positive}}\,{\text{control}}}} \times 100{\text{\% ,}} $$

where the *A*412(*t*10 − *t*0)test compound is the change in the absorbance at *A*412 nm between 0 and 10 min for the test compound, the *A*412(*t*10 − *t*0)positive control is the change in the absorbance at A412 nm between 0 and 10 min for the CPO sample, and the *A*412(*t*10 − *t*0)solvent control is the change in the absorbance at *A*412nm between 0 and 10 min for the 0.2% ethanol sample.

The concentration-dependent human and rat AChE inhibition curves were analysed to define the half maximal inhibitory concentrations (IC50) for both DZO and DZN using non-linear regression, dose response-inhibition-variable, log(inhibitor) vs. response-variable slope (four parameters) in GraphPad Prism 5, version 5.04 (GraphPad software, San Diego California USA), with 95% confidential interval. To further define whether the concentration-dependent DZN and DZO AChE inhibition curves in both rat and human were parallel or not, the hillslope values of these curves were statistically compared using non-linear regression, Dose Response-Inhibition-Variable, log(inhibitor) vs. response-variable slope (four parameters), compare tab “Do the best fit values of selected parameters differ between data sets” in GraphPad Prism 5, version 5.04 (GraphPad software, San Diego California USA) based on instruction from GraphPad (GraphPad 2009).

### Determination of unbound fraction of DZO and DZN in the in vitro medium and in vivo

A previous study from Heilmair et al. (2008) showed that the presence of a low level of BSA (0.1 mg/ml) will not significantly affect the free concentration of OP chlorpyrifos-oxon in solution. Based on this observation, the in vitro unbound fraction of both DZN (fuDZN_in vitro_) and DZO (fuDZO_in vitro_) in the in vitro medium are set at 1. To assess whether this assumption is reasonable, the fuDZO_in vitro_ was determined. This was done using rapid equilibrium dialysis (RED) performed in line with the manufacturer’s protocol (Thermo Fisher Scientific 2012). To this end, 200 µl in vitro medium containing 0.05 µM DZO were added to the sample chamber and 350 µl PBS buffer consisting of 100 mM sodium phosphate and 150 mM sodium chloride to the buffer chamber, separated by a semipermeable membrane in the RED insert device. The whole device was then incubated for 5 h at 37℃ on a shaker at 250 rmp to reach equilibrium. Then 50 µl of post-incubation sample were separately collected from the sample and buffer chambers into the corresponding Eppendorf tubes. After this 50 µl of PBS buffer was added to the sample taken from the sample chamber and 50 µl of in vitro medium was added to the sample taken from buffer chamber and subsequently 300 µl ice-cold 90% acetonitrile (ACN/water, v/v) were added to both samples to precipitate the protein. All samples were put on ice for 30 min, followed by centrifugation for 30 min at 15,000*g*. The supernatants were collected for LC–MS/MS analysis of the amount of DZO. The fuDZO_in vitro_ was calculated by dividing the concentration of DZO in the buffer chamber by the concentration DZO in the sample chamber.

Given that only the free concentration of DZN and DZO can inhibit AChE in vivo, the fraction of unbound DZN in vivo (fuDZN_in vivo_) and of unbound DZO in vivo (fuDZO_in vivo_) were estimated based on SMILES string of compound using the pkCSM prediction tool (Pires et al. 2015; pkCSM 2020).

### PBK model for DZN

**Fig. 2:**
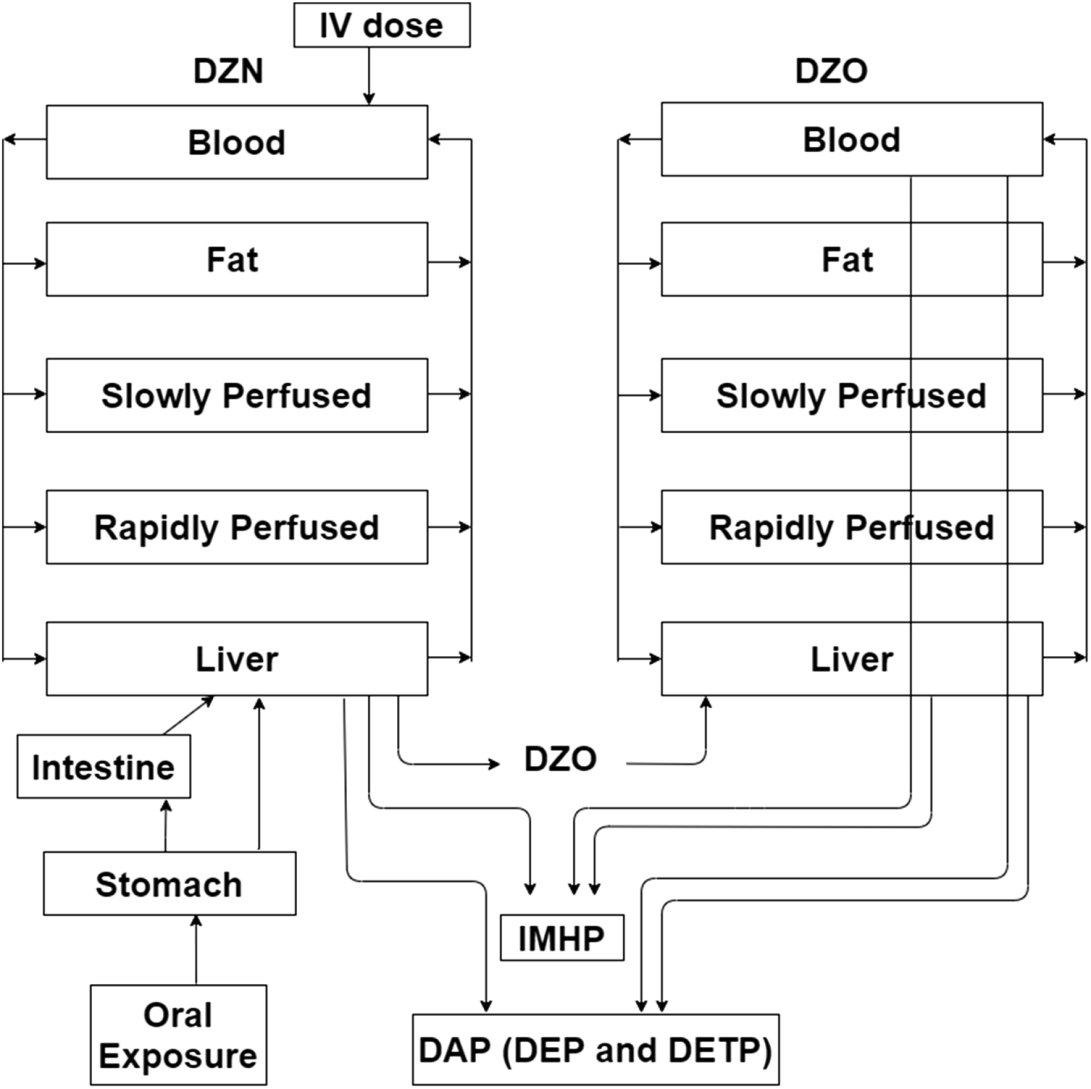
Structure of the PBK model for DZN in rat and human with a submodel for DZO

The PBK model for DZN was developed based on the model from Poet et al. (2004) with some modifications for both rat and human. The model structure is presented in Fig. 2. The model contains a submodel for the metabolite DZO and was defined to include compartments for rapidly perfused tissue, slowly perfused tissue, liver, fat and blood for both the parent compound DZN and its metabolite DZO. The model also contains a compartment to describe the urinary elimination of the metabolites IMHP and DAP (sum of DEP and DETP). In the currently developed model, oral exposure was included since we aimed at defining a POD for risk assessment of exposure via food and drinking water, and intravenous (IV) exposure was included for model evaluation. The fractional absorption (fa) was set equal to literature reported values, being 0.8 in rat (Poet et al. 2004) and 0.66 in human (Garfitt et al. 2002) and applied to the overall dose. The absorption of DZN from the stomach into the liver was described using a two-compartment gastrointestinal tract model as reported by Poet et al. (2004), with a first-order rate constant for absorption of DZN from the stomach into the liver (KaS) of 0.1 /h for rat and 0.32/h for human (Poet et al. 2004), a first-order rate constant for transfer of DZN from the stomach into the intestine (KsI) of 0.48/h for both rat and human, a first-order rate constant for absorption DZN from the intestine into the liver (KaI) of 0.59/h for both rat and human, (Poet et al. 2004), and Ke values for the elimination of IMHP or DAP (sum of DEP and DETP) into the urine amounting to Ke IMHP = 0.29/h, Ke DEP = 0.29 /h and Ke DETP = 0.29/h for rat (Poet et al. 2004), and Ke IMHP = 12/h, Ke DEP = 12 /h and Ke DETP = 12/h for human (Garfitt et al. 2002; Poet et al. 2004).

The physiological parameters for rat and human were obtained from Brown et al. (1997) as well as Gearhart et al. (1990), and are summarised in Table 1. The partition coefficients for both DZN and DZO were obtained using the approach described by DeJongh et al. (1997), based on the value Log*K*_ow_ which was derived from clogP estimated using ChemDraw professional 16.0 (Cambrigesoft) (Table 1). The kinetic parameters for biotransformation of DZN in rat and human were obtained by conducting in vitro liver microsomal/plasma incubations as described in the section “In vitro metabolic incubations for deriving kinetic parameters”. The bioactivation and detoxification of DZN by CYP450 were assumed to occur only in the liver (Poet et al. 2004), and the resulting DZO was transferred to the DZO submodel. In the current model, only unbound DZN and DZO are assumed to be metabolised. The first-pass metabolism of DZN by the intestine was not taken into account in the current model because the metabolic conversion in the intestine derived from the conversion in incubations with intestinal microsomes upon scaling to the whole organ, appeared less than 5% of that obtained in a similar way for the liver (data not shown). Since PON1 can be expected to be present in both liver and blood, the PON1-mediated detoxification of DZO was modelled to occur in these two compartments (Pyati et al. 2015). To scale the in vitro Vmax values to the in vivo situation, the following scaling factors were used; 35 mg microsomal protein/g liver for rat hepatic metabolism (Medinsky et al. 1994), 32 mg microsomal protein/g liver for human hepatic metabolism (Barter et al. 2007), 77 mg plasma protein/ml plasma (total plasma protein concentration) for human plasma metabolism, and 60 mg plasma protein/ml plasma (total plasma protein concentration) for rat plasma metabolism. Plasma volume in rat and human were assumed as 55% of their corresponding blood volume (O'Neil 1999). The *K*_m_ values determined in vitro were assumed to be equal to in vivo *K*_m_ values. When lacking experimental values the blood plasma ratio (B/P) is often assumed to be 1 for basic compounds or 0.55 (1-haematocrit) for acidic compounds (Cubitt et al. 2009). In the current study, because experimental data were not available, and both DZN and DZO are basic compounds, the B/P ratio of DZN and DZO were assumed to be 1. Therefore, no correction was required between blood and plasma concentration.

**Table 1:** Summary of physiological and physicochemical parameters for the PBK models for DZN and its metabolite DZO in rat and human (Brown et al. 1997; DeJongh et al. 1997; Gearhart et al. 1990)

Model parameters	Rat		Human
*Physiological parameters*			
Body weight (BW; kg)	0.25		70.0
*Percentage of body weight*			
Liver	3.4		2.6
Fat	7.0		21.4
Rapidly perfused	4.8		5.4
Slowly perfused	66.7		58.0
Blood	7.4		7.9
*Flow (l/hr/kg BW*^*0.74*^*)*			
Cardiac output	15.0		15.0
*Percentage of cardiac output*			
Liver	25.0		22.7
Fat	9.0		5.2
Rapidly perfused	51.6		43.0
Slowly perfused	14.4		29.1
*Tissue: blood partition coefficients for DZN*			
Liver	13.7		7.1
Fat	211.0		137.7
Rapidly perfused	13.7		7.1
Slowly perfused	8.7		4.5
*Tissue: blood partition coefficients for DZO*			
Liver	3.9		2.6
Fat	89.6		72.4
Rapidly perfused	3.9		2.6
Slowly perfused	2.4		1.9

Because previously it was shown that not only DZO but also its parent compound DZN are able to inhibit AChE (Li et al. 2019), in the present study both DZN and DZO were considered to be able to act as AChE inhibitor. To determine the combined effect of DZN and DZO at the target site, the free effective blood maximum concentration of DZN and DZO was expressed in DZO equivalents using a toxic equivalency factor (TEF) (see Eq. (3):

3 $$ {\text{Total}}\,{\text{free}}\,{\text{in}}\,{\text{vivo}}\, {\text{DZN and}}\, {\text{DZO}}\,{\text{concentration}}\,{\text{expressed}}\,{\text{in}}\,{\text{DZO}}\,{\text{equivalents}} = [{\text{DZN]}} \times {\text{fuDZN}}\,{\text{in}}\,{\text{vivo}} \times {\text{TEFDZN}} + \left[ {{\text{DZO}}} \right] \times {\text{fuDZO}}\,{\text{in}}\,{\text{vivo}} \times {\text{TEFDZO}}. $$

In which the Total free in vivo DZN and DZO concentration expressed in DZO equvalents represents the free blood maximum concentration of DZO plus DZN expressed in DZO equivalents using the TEF values for DZO and DZN, [DZN] and [DZO] represent the total blood maximum concentrations of DZN and DZO, which were corrected to their corresponding free internal maximum concentrations using their unbound fraction in vivo fuDZN_in vivo_ and fuDZO_in vivo_, and the TEFDZN and TEFDZO are the toxic equivalency factors of DZN and DZO. The TEF for DZO was set at 1.0 and the TEF for DZN was defined using Eq. (4) based on its IC50 and the IC50 of DZO for inhibition of rat AChE or human hrAChE for the rat and human model, respectively.

4 $$\mathrm{TEF DZN }=\frac{\mathrm{IC}50\mathrm{ DZO }}{\mathrm{IC}50\mathrm{ DZN }}.$$

The free internal maximum concentration of DZN plus DZO expressed in DZO equivalents was subsequently used to extrapolate the in vitro AChE concentration–response curve of DZO to its corresponding in vivo DZN-dose response curve using reverse dosimetry (see below).

All differential equations and the mass balance were coded in Berkeley Madonna software version 8.3.18 ((Macey and Oster, UC Berkeley, California) using Rosenbrock’s algorithm for stiff systems. The full model code is presented in Supplementary data II.

### Sensitivity analysis

The key parameters that have the largest influence on the prediction of the model parameter of interest, being the maximum combined DZN and DZO free blood concentration expressed in DZO equivalents was identified by performing a sensitivity analysis at low non-toxic dose levels of DZN of 3 mg/kg bw (rat) and 0.011 mg/kg bw (human), and at high-DZN dose levels of 300 mg/kg bw (rat) and 293 mg/kg bw (human), the latter two dose levels representing toxic dose levels reported for rat and human respectively (JMPR 2016; Poklis et al. 1980). The normalized sensitivity coefficients (SC) were calculated based on Eq. (5):

5 $$ {\text{SC}} = \frac{{\left( {C^{\prime} - C} \right)}}{{(P^{^{\prime}} - P)}} \times \left( \frac{P}{C} \right). $$

In which *P* represents the original parameter value in the PBK model and *P′* is the parameter value with a 5% increase, while *C* is the model output with the original parameter values and *C*′ is the model output with a parameter value with an increase of 5%. Each parameter was analyzed individually while other parameters were kept at their initial value.

### Model evaluation

The developed rat DZN model was evaluated by comparing predicted time-dependent plasma concentrations of DZN (both upon oral and intravenous IV administration) with their corresponding available in vivo data (Lu et al. 2003; Poet et al. 2004; Wu et al. 1996). The performance of the human DZN model was assessed by comparing the predicted urinary DAP excretion against available in vivo data (Garfitt et al. 2002).

### Translation of the in vitro concentration response curve to an in vivo dose response curve

In this step, the species-specific in vitro DZO AChE inhibition concentration–response curves were converted to the corresponding DZN dose–response curves via reverse dosimetry using the rat or human TEF-coded PBK models. To this end, the in vitro DZO concentrations were assumed to be equal to the nominal concentrations given that the fuDZO_in vitro_ was considered equal to 1. Subsequently, the DZO concentration in vitro was set equal to the free maximum DZN and DZO concentration expressed in DZO equivalents by multiplying with TEFDZO, to determine the DZN dose that would result in this concentration, leading to the corresponding inhibition, ultimately generating the predicted DZN dose–response curve for in vivo RBC AChE inhibition in rat and human by DZN exposure. This was done using Eq. (6):

6 $$\mathrm{Total free in vivo DZN and DZO concentration expressed in DZO equivalents }=\left[\mathrm{DZO}\right]\mathrm{in vitro}\times \mathrm{fuDZO in vitro}\times \mathrm{TEFDZO}$$

The correction for protein binding in vivo was done as described above (see Eq. 3).

### Determination of a point of departure (POD) based on the predicted in vivo dose–response curve

The predicted in vivo RBC AChE inhibition dose–response curves obtained for rat were validated against available in vivo data (JMPR 2016; USEPA 2016), and subsequently used to derive a POD for evaluation of the acute toxicity upon oral exposure using a BMD analysis. In the present study, the BMDL_10_ was used as POD since also the USEPA used the BMDL_10_ as POD to define the acute reference dose (ARfD) (USEPA 2016). To obtain the BMDL_10_, the Benchmark Dose Software version 3.1.2 (USEPA 2020) was used. Of all available models (Exponential, Hill, Power, Linear and Polynomial) for fitting of continuous data, only the Exponential and Hill models provided adequate fits to the data, and were employed for derivation of predicted BMDL_10_ values for rat or human with BMR type of Std. Dev, confidential level of 0.95, distribution type of normal and variance type of constant. The BMDL_10_ value with the lowest AIC was chosen as POD. Finally, the obtained POD values were evaluated against reported BMDL_10_ values or POD values from EFSA (2006), JMPR (2016) and USEPA (2016).

## Results

### Kinetic data and total protein concentration

**Fig. 3:**
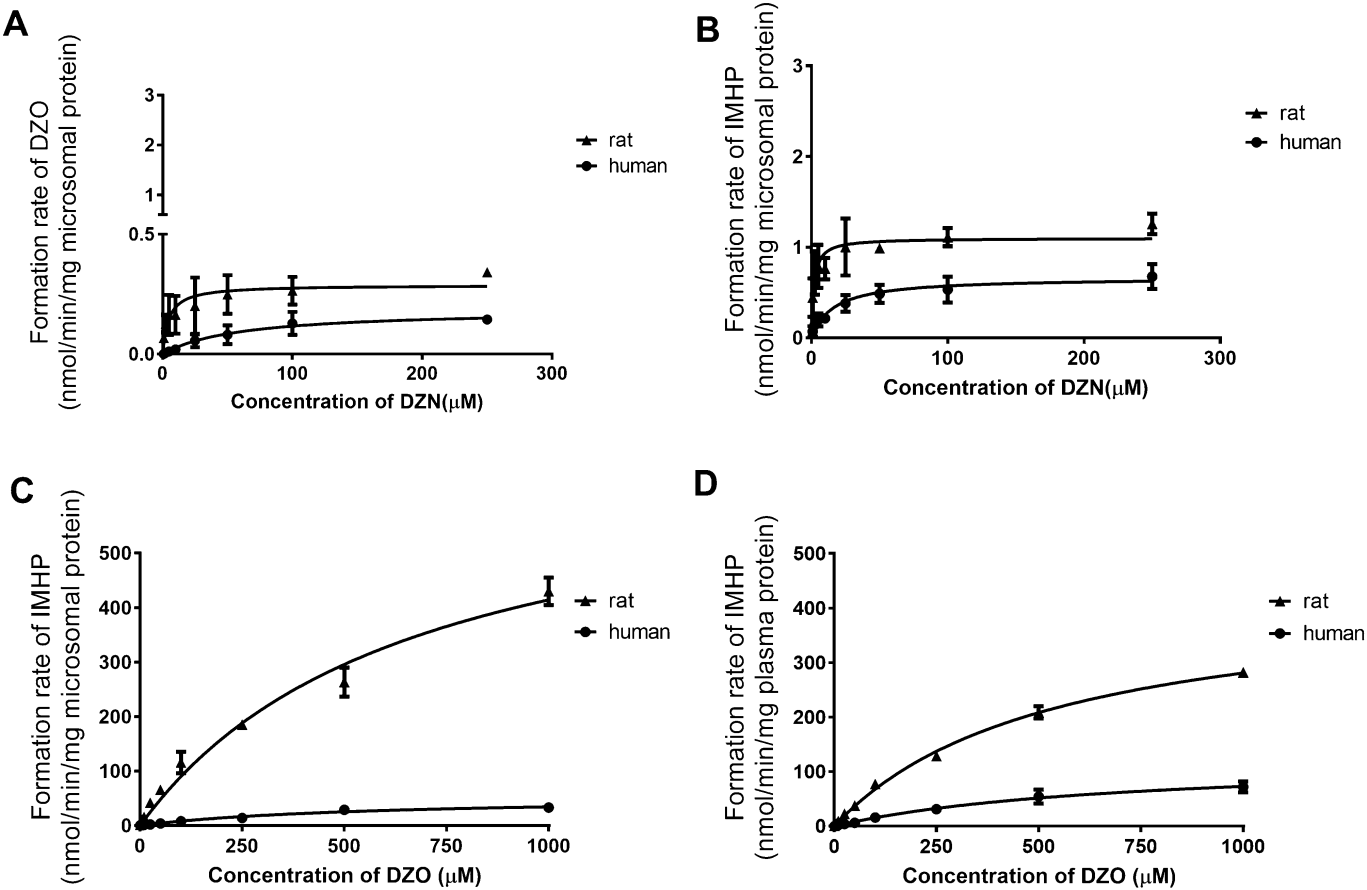
CYP450-mediated DZN concentration-dependent formation of **a** DZO and **b** IMHP in incubations with pooled rat (filled triangle) or human (filled circle) liver microsomes, and PON1-mediated DZO concentration-dependent formation of IMHP in incubations with **c** pooled rat (filled triangle) or human (filled circle) liver microsomes, or **d** pooled rat (filled trangle) or human (filled circle) plasma. Data points represent mean ± SD of two experiments for each conversion

The kinetic parameters for biotransformation of DZN and DZO by the different pathways were determined by incubating increasing concentrations of DZN with pooled human or rat liver microsomes, and DZO with pooled human or rat liver microsomes or plasma (Fig. 3). The apparent *V*_max_, *K*_m_ and the catalytic efficiency (calculated as *V*_max_/*K*_m_) derived from these data, as well as the determined total protein concentration of plasma are shown in Table 2. In general, for both rat and human, the CYP450-mediated detoxification of DZN to IMHP is faster than its CYP450-mediated bioactivation to DZO. The PON1-mediated detoxification of DZO in liver and plasma was even faster and more efficient. Together these data indicate that detoxification is preferred over bioactivation in both rat and human. Comparison of the data for rat and human, reveals that the unscaled catalytic efficiency of CYP450-mediated bioactivation is 20-fold higher in rat than human while detoxification is 15-fold more efficient in rat compared to human. These differences originate from a 12.6-fold lower *K*_m_ for the CYP450-mediated bioactivation reaction, and a 9.3-fold lower *K*_m_ for CYP450-mediated detoxification in rat than in human. PON1-mediated conversion of DZO in rat liver and plasma was 10.7-fold and 4.5-fold faster than that in human liver and plasma, respectively, due to a 12.9-fold higher Vmax in rat liver and 3.5-fold higher Vmax in rat plasma as compared to human plasma. Besides, the results also show that rat plasma and human plasma have comparable total protein concentrations, being 60 mg/ml in rat plasma and 77 mg/ml in human plasma (Table 2).

**Table 2:** Kinetic parameters for biotransformation of DZN and DZO in liver and plasma

Pathway	Rat	Human
**Liver**		
**DZN to DZO**		
Vmax (nmol/min/mg microsomal protein)	0.288	0.187
Km (µM)	4.745	59.600
CE (ml/min/mg microsome protein)^a^	0.060	0.003
**DZN to IMHP**		
Vmax (nmol/min/mg microsomal protein)	1.098	0.665
Km (µM)	1.764	16.340
CE (ml/min/mg microsome protein) ^a^	0.620	0.041
**DZO to IMHP**		
Vmax (nmol/min/mg microsomal protein)	691.100	53.490
Km (µM)	668.000	557.400
CE (ml/min/mg microsome protein) ^a^	1.030	0.096
**Plasma**		
**DZO to IMHP**		
Vmax (nmol/min/mg plasma protein)	431.400	124.000
Km (µM)	535.600	701.000
CE (ml/min/mg plasma protein) ^a^	0.810	0.180
Total protein concentration (mg/ml)	60.000	77.000

^a^CE = catalytic efficiency (ml/min/mg microsome protein or ml/min/mg plasma protein) calculated as *V*_max_/*K*_m_

### AChE inhibition concentration–response curve and TEF calculation

**Fig. 4:**
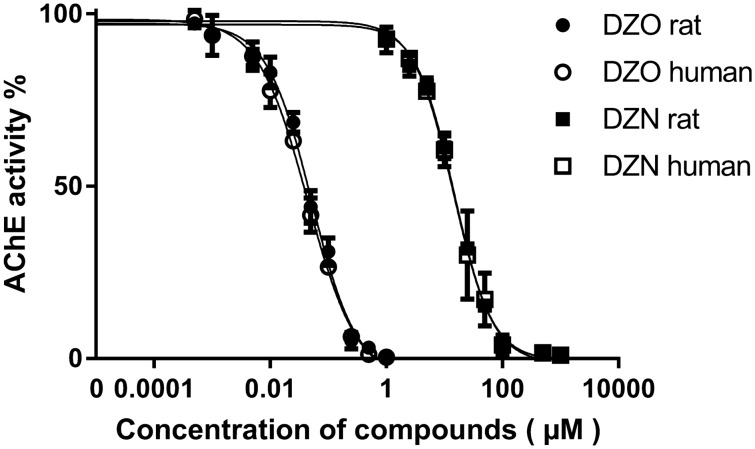
Effect of increasing concentration of DZO (circle) and DZN (square) on acetylcholinesterase (AChE) activity of rat (filled circle or square) and human (unfilled circle or square) at 37 ℃. Each value represents the mean ± SD of three independent experiments

Figure 4 shows the DZO concentration-dependent inhibition of rat and human AChE activity, with 50% inhibition (IC50) being observed at a concentration of 0.0515 µM (with the 95% confidence interval ranging from 0.0443 to 0.0610 µM) for rat and 0.0440 µM (with the 95% confidence interval ranging from 0.0380 to 0.0521 µM) for human. The IC50 values observed for DZN were substantially higher amounting to 14.66 µM (with the 95% confidence interval ranging from 12.91 to16.63 µM) for rat and 14.26 µM (with the 95% confidence interval ranging from 11.64 to17.59 µM) for human (Fig. 4). These IC50 values indicate that rat and human AChE appear to show comparable sensitivity towards in vitro inhibition following DZO and DZN exposure. Besides, the comparison of hillslope values of the curves indicated that the DZN and DZO curves are parallel for both human and rat (the hillslope value of AChE inhibition induced by DZN and DZO in human is − 1.2700, and − 1.0200, with *p* value of 0.0797, and that in rat is − 1.3220 and − 1.1240, with *p* value of 0.1504. To incorporate the TEF method into the PBK model developed for predicting the combined free blood concentration of DZN and DZO at the target site (RBC AChE) in DZO equivalents, the TEF value for DZN was calculated based on the IC_50_ values derived from the in vitro concentration-AChE inhibition curves, setting the TEF of DZO at 1.0. The TEF values thus obtained for DZN and DZO are 0.00351 and 1.0 in rat, and 0.00310 and 1.0 in human.

### PBK model validation

**Fig. 5:**
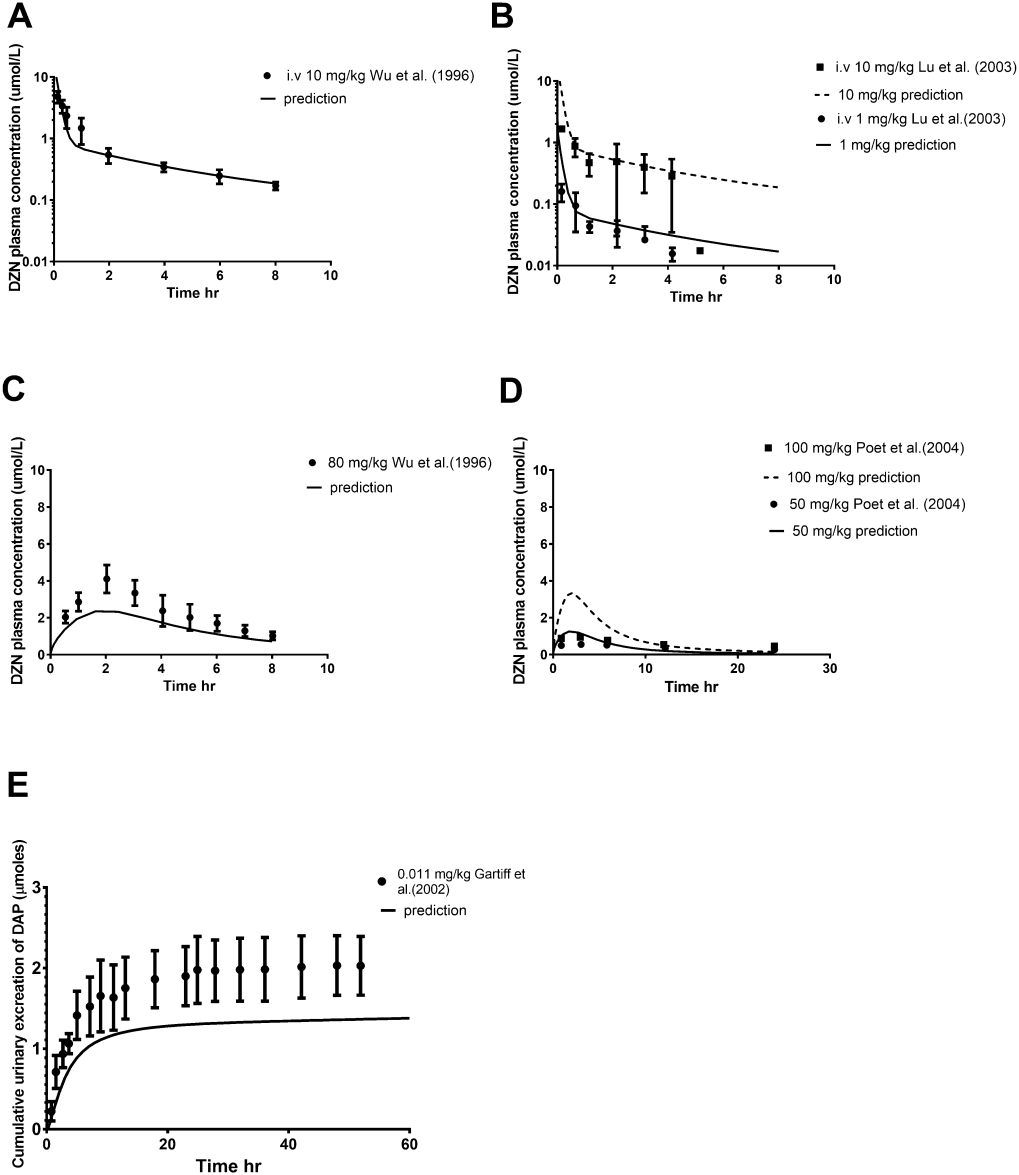
Comparison between reported in vivo data and PBK model predictions for **a** the time-dependent DZN plasma concentration in rats upon IV administration of DZN at 10 mg/kg bw (Wu et al. 1996); **b** the time-dependent DZN plasma concentration in rats upon IV administration of DZN at 1 mg/kg bw and 10 mg/kg bw (Lu et al. 2003); **c** the time-dependent DZN plasma concentration in rats upon oral administration of DZN at 80 mg/kg bw (Wu et al. 1996); **d** the time-dependent DZN plasma concentration in rats upon an oral DZN dose of 50, and 100 mg/kg bw (Poet et al. 2004); **e** the urinary excretion of DAP in humans upon an oral dose of 0.011 mg/kg bw (Garfitt et al. 2002)

The PBK models developed for DZN were evaluated against in vivo data. For the rat model, the evaluation was based on comparison of the model predictions with four sets of available in vivo data. These included: (1) the time-dependent DZN plasma concentration upon IV administration of DZN at 10 mg/kg bw (Fig. 5a) (Wu et al. 1996); (2) the time-dependent DZN plasma concentration upon IV administration of DZN at 1 and 10 mg/kg bw (Fig. 5b) (Lu et al. 2003); (3) the time-dependent DZN plasma concentration upon oral administration of DZN at 80 mg/kg bw (Fig. 5c) (Wu et al. 1996); and (4) the time-dependent DZN plasma concentration upon an oral DZN dose of 50, and 100 mg/kg bw (Fig. 5d) (Poet et al. 2004). The data reveal that the model adequately predicts the DZN plasma concentration upon IV administration (Fig. 5a, b). Upon oral dosing, the predictions vary, but this seems to be also related to differences in the experimental data. The DZN plasma levels reported by Poet et al. (2004) for example are around seven-fold and four-fold lower at a dose level of 50 and 100 mg/kg bw than what is reported by Wu et al. (1996) at a dose level of 80 mg/kg bw, pointing at a discrepancy in between these in vivo data. In the present study, the PBK model predictions matched the reported plasma DZN levels from Wu et al. (1996) well based on the acceptance criteria from the WHO (WHO 2010) (predictions are between 0.8- to two-fold different from in vivo data) (Fig. 5c), while the predictions are two- to three-fold different from the values reported by Poet et al. (2004) (Fig. 5d). Given that the PBK model accurately predicted the data from the Wu et al. (1996) (Fig. 5c), and the fact that the maximum plasma concentration levels reported by Poet et al. (2004) at dose levels of 50 and 100 mg/kg bw are seven-fold and four-fold lower than the concentration level reported by Wu et al. (1996) at 80 mg/kg bw (instead of only 1.6-fold lower and 1.25-fold higher in line with the dose differences), it is concluded that these lower values reported by Poet et al. (2004) might be related to an experimental factor resulting from for example lower oral bioavailability of the administered dose. Based on these considerations it was concluded that the PBK model was acceptable for further reverse dosimetry. This is further supported by the data presented in Fig. 5e show that the related human PBK model well predicted urinary excretion of DAP (within 1.5-fold difference compared with DAP urinary excretion data in human) upon an oral dose of 0.011 mg/kg bw given to human subjects (four men and one woman, age range 30–50 years, weight range 76–90 kg) by Garfitt et al. (2002).

### Sensitivity analysis

**Fig. 6:**
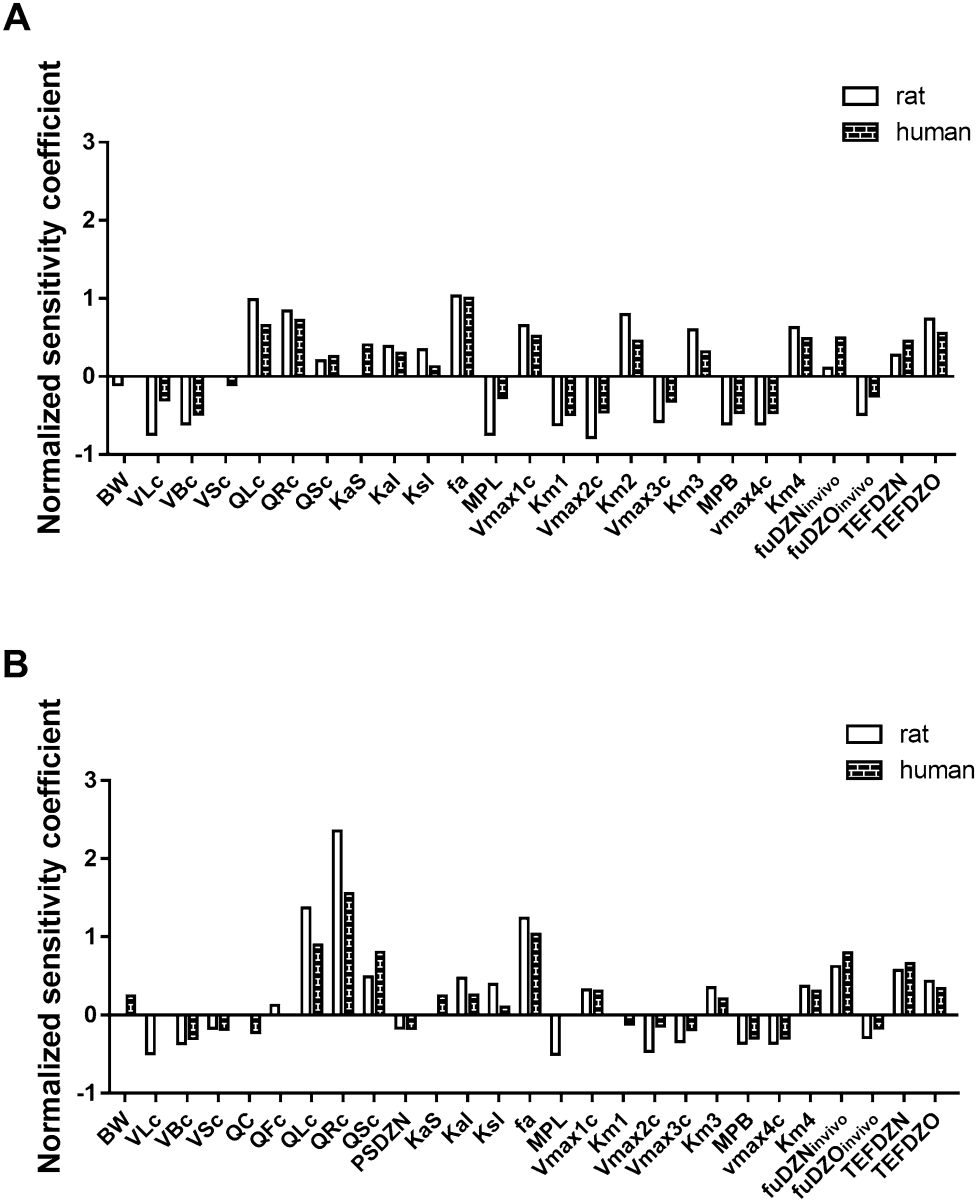
Sensitivity analysis for the predicted free blood concentration of DZN plus DZO expressed in DZO equivalents at **a** low dose levels of DZN of 3 mg/kg (rat) and 0.011 mg/kg bw (human), and **b** high dose levels of DZN of 300 mg/kg (rat) and 293 mg/kg bw (human). The parameters represent: *BW* body weight, *VLc* fraction of liver tissue, *VBc* fraction of blood, *VSc* fraction of slowly perfused tissue (bone, muscle and skin), *QC* cardiac output, *QFc* fraction of blood flow to fat, *QLc* fraction of blood flow to liver, *QRc* fraction of blood flow to richly perfused tissue, *QSc* fraction of blood flow to slowly perfused tissue (muscle, skin, bone), *PSDZN* slowly perfused tissue/blood partition coefficient of DZN, *KaS* first-order rate constant for absorption DZN from stomach into liver, *KaI* first-order rate constant for absorption DZN from intestine into the liver, *KsI* first-order rate constant for transfer of DZN from stomach to intestine, *fa* fractional absorption, *MPL* liver microsomal protein yield, *V*_*max1c*_ maximum rate for conversion of DZN to DZO, *K*_*m1*_ Michaelis Menten constant for conversion of DZN to DZO, *V*_*max2c*_ maximum rate for conversion of DZN to IMHP, *K*_*m2*_ Michaelis Menten constant for conversion of DZN to IMHP, *V*_*max3c*_ maximum rate for conversion of DZO to IMHP in liver, *K*_*m3*_ Michaelis Menten constant for conversion of DZO to IMHP in liver, *V*_*max4c*_ maximum rate for conversion of DZO to IMHP in plasma, *K*_*m4*_ Michaelis Menten constant for conversion of DZO to IMHP in plasma, *MPB* plasma protein scaling factor, *fuDZN*_*in vivo*_ free fraction of DZN in vivo, *fuDZO*_*in vivo*_ free fraction of DZO in vivo, *TEFDZN* toxic equivalency factor of DZN, *TEFDZO* toxic equivalency factor of DZO

In the present study, the impact of each parameter on the model output (the maximum free blood concentration of DZO plus DZN expressed in DZO equivalents) was determined by performing a sensitivity analysis. Only the parameters with normalized sensitivity coefficient higher than 0.1 (absolute value) are shown in Fig. 6.

For both rat and human, the predicted maximum blood free concentration of DZN plus DZO expressed in DZO equivalents is substantially influenced by body weight, kinetic parameters for all pathways of DZN, volume of liver, volume of blood, blood flow to rapidly and slowly perfused tissue as well as to liver tissue. In addition, all three absorption rate constants, fraction of dose absorbed, liver microsomal protein yield scaling factor, plasma protein scaling factor, the fuDZN_in vivo_, fuDZO_in vivo_ as well as the TEFDZN and TEFDZO appear to have a substantial influence on the prediction at low dose of DZN. At high dose level, similar results were obtained except for the influence of the kinetic parameters and plasma total protein concentration that became less influential, while slowly perfused tissue/blood partition coefficient of DZN and cardiac output started to play a role.

### Unbound fraction of DZO and DZN in the in vitro medium and in vivo

The fuDZN_in vivo_ and fuDZO_in vivo_ were predicted to be 0.329 and 0.302, respectively. An fuDZO_in vitro_ value of 0.96 was obtained (with the recovery rate of post-dialysis of DZO in the in vitro medium being 74%), indicating that the presence of a low level (0.1 mg/ml) of BSA in the in vitro medium does not substantially affect the free fraction of DZO. This observation is in line with the data reported by Heilmair et al. (2008) as mentioned above. A similar result is expected for DZN since DZN and DZO have comparable unbound fractions in plasma. Therefore, the unbound fraction of both DZN and DZO in vitro was set at 1.

### Relative contribution of DZN and DZO to plasma DZO equivalents

**Fig. 7:**
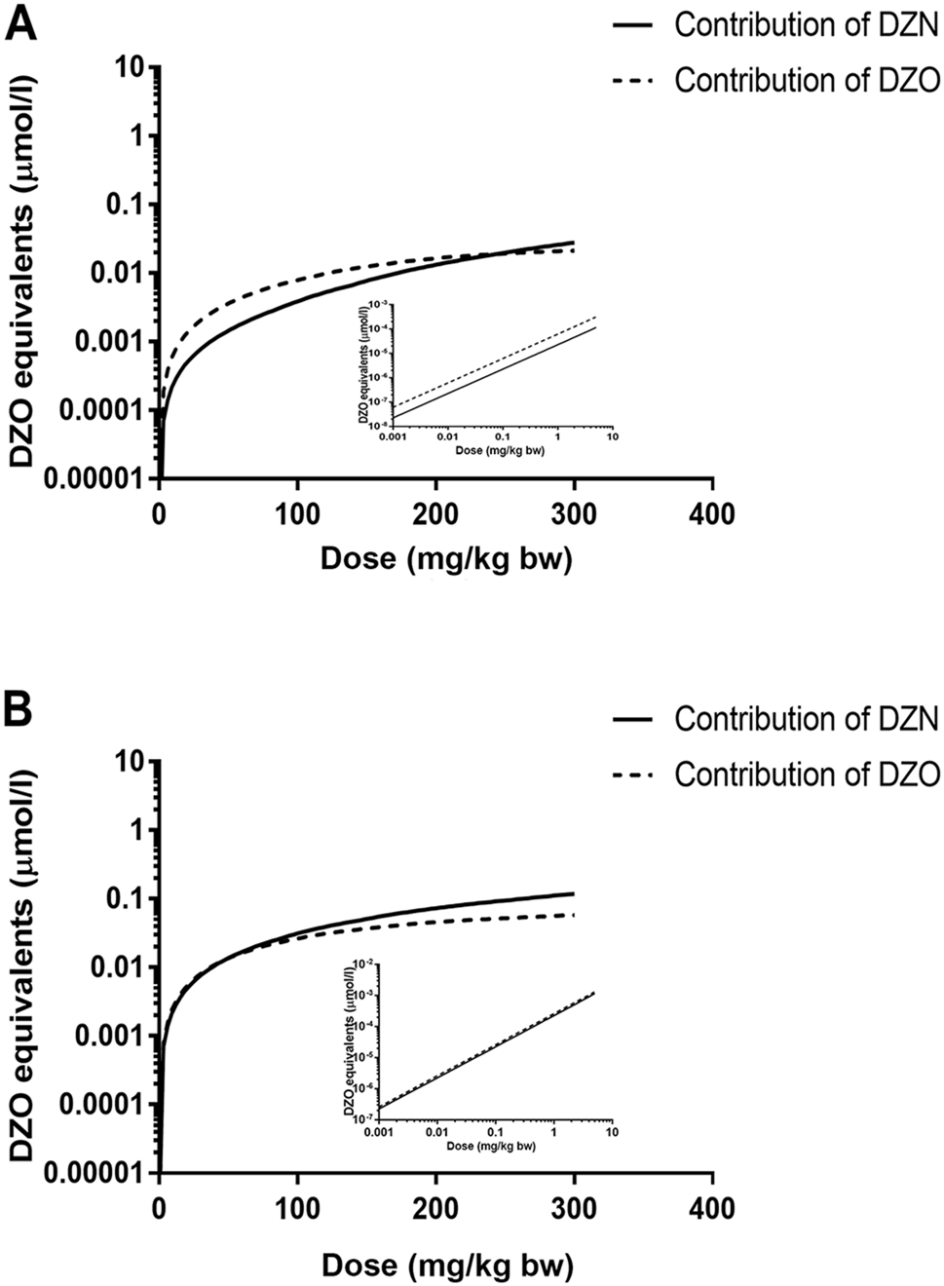
The PBK model-based predicted dose-dependent relative contribution of DZN and DZO to the free blood concentration expressed in DZO equivalents in **a** rat and **b** human. The insert presents the data at the lower dose levels (up to 10 mg/kg bw) in some more detail

Figure 7 shows the predicted dose-dependent relative contribution of DZN and DZO to the total free blood concentration expressed in DZO equivalents in rat and human. These results indicate that, apart from DZO, DZN is predicted to be another major contributor to the blood DZO equivalents, in spite of its relatively low TEF values of 0.00351 in rat and 0.00310 in human. This is especially apparent at high dose levels (at dose levels higher than 240 mg/kg in rat and 50 mg/kg in human), the role of DZN even outweighs that of its active metabolite DZO because of its high concentration and the almost saturation of its conversion to DZO. Similarly, the predicted free blood maximum concentration of DZO for a DZN dose range from 0 up to 300 mg/kg bw (supplementary data III) in humans is around 2- to 4-fold higher than that in rats, while this inter-species difference increases to around 4- to 10-fold when comparing the predicted dose-dependent maximum blood concentrations expressed in DZO equivalents in human and rat, with the values in human being higher (supplementary data III). Overall, Fig. 7 and supplementary data III together reflect the relatively higher contribution of DZN to the toxicity in human than in rats, and also that it is essential to take the contribution of both DZN and DZO into account.

### Predicted in vivo dose–response curves for AChE inhibition and their evaluation

**Fig. 8:**
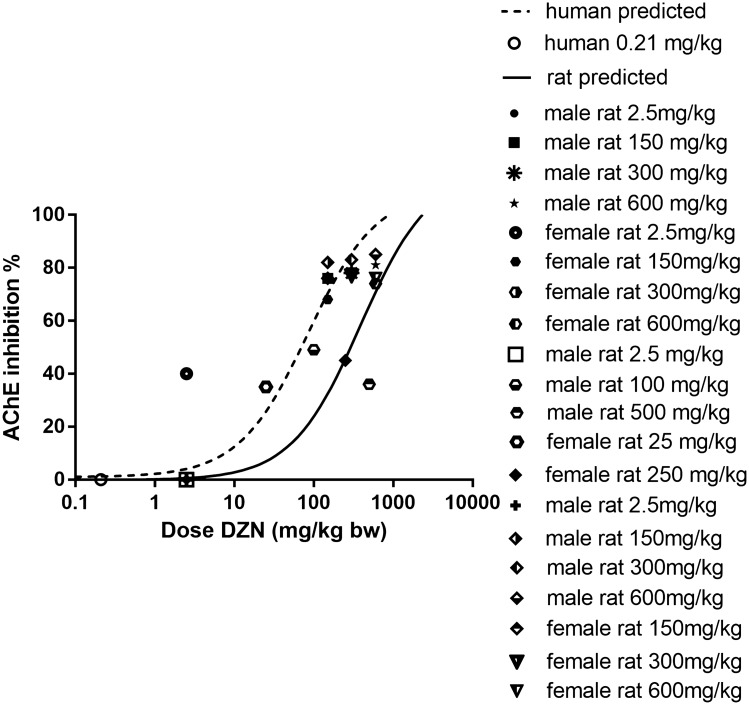
The predicted in vivo dose–response curves for AChE inhibition upon DZN exposure in rat (solid line) and human (dashed line) using PBK model-based reverse dosimetry. The individual data points represent available in vivo data for RBC AChE inhibition in rat and human upon oral exposure to DZN at different dose levels as reported by JMPR (2016) and USEPA (2016)

Figure 8 presents the predicted in vivo dose–response curves for AChE inhibition upon DZN exposure in rat and human. Based on the predicted dose–response curves obtained, human seems more sensitive than rat in terms of AChE inhibition caused by DZN exposure, although a similar intrinsic potency was found from the in vitro concentration–response curves for human and rat AChE inhibition from which these in vivo predicted curves were derived (Fig. 4). This indicates that differences in kinetics influence the inter-species differences in in vivo sensitivity.

For comparison, Fig. 8 also presents the individual data points for AChE inhibition as reported by JMPR and USEPA (JMPR 2016; USEPA 2016). Comparison of these data to the predicted curves reveals that the predictions are in line with the reported in vivo data.

### Predicted BMDL_10_ values and their evaluation

The dose–response curves obtained were used to derive BMDL_10_ values for both rat and human allowing comparison to PODs available from previous evaluations (EFSA 2006; JMPR 2016; USEPA 2016). The predicted BMDL_10_ values amounted to 2.1 and 12.6 mg/kg bw for human and rat respectively (Table 3). The BMDL_10_ value for human was 6-fold lower than that for rat, a difference that is lower than the default uncertainty factor of 10 for inter-species differences, providing support for a chemical-specific adjustment factor. The BMDL_10_ of 12.6 mg/kg bw for rat, compares well to the BMDL_10_ for inhibition of brain and RBC AChE in male adult rats upon oral DZN administration, amounting to 12.175 mg/kg bw/day for inhibition of brain AChE and 4.804 mg/kg bw/day for inhibition of RBC AChE, respectively (USEPA 2016). The predicted BMDL_10_ of the present study for rat RBC AChE inhibition appears the same as the BMDL_10_ reported for inhibition of brain AChE and 2.6-fold higher than that for inhibition of RBC AChE. However, EPA used a BMDL_10_ value of 3 mg/kg bw derived from RBC AChE inhibition in female rat pups (PND11) (USEPA 2016) as the POD for deriving an ARfD. A previous study indicated that the inhibition of brain AChE in pups at postnatal day 17 was 2-fold higher than in adult rats (75% instead of 38%) at a similar oral dose of 75 mg/kg bw (Padilla et al. 2004), suggesting that the early life stage of rat (pups) show a greater sensitivity than adult rats. Therefore the predicted BMDL_10_ in the current study for adult rats can be further corrected by this factor 2, resulting in a POD of 6.3 mg/kg bw which is 2.1-fold higher than the POD of 3 mg/kg bw used by the EPA (USEPA 2016).

**Table 3:** Comparison of predicted BMDL_10_ values to reported BMDL_10_ values and to the NOAEL values established by USEPA (2016), EFSA (2006) and JMPR (2016) for DZN

PODs	Reported NOAEL from EFSA and JMPR (adult rat) (mg/kg bw)	Reported BMDL_10_ from EPA (adult rat) (mg/kg bw)	Reported BMDL_10_ from EPA (pups) (mg/kg bw	Predicted BMDL_10_ for adult rat (mg/kg bw)	Predicted BMDL_10_ for rat pups (mg/kg bw)	Predicted BMDL_10_ for human (mg/kg bw)
Diazinon	2.5	4.8	3.0	12.6	6.3^a^	2.1

^a^Value derived from the BMDL_10_ for adult rats taking into account the results from an in vivo study (Padilla et al. 2004), showing that pups are expected to be 2-fold more sensitive than adults

Different from the EPA, JMPR used a no-observed-adverse-effect level (NOAEL) of 2.5 mg/kg bw from an acute (neuro)toxicity study of DZN in rats (JMPR 2016) based on inhibition of brain and RBC AChE activity in female rat at a lowest observed adverse effect level (LOAEL) of 25 mg/kg bw. Similarly, EFSA used an overall NOAEL of 2.5 mg/kg bw from three rat studies (EFSA 2006) based on AChE inhibition alone or AChE inhibition together with reversible neurotoxic effects occurring at a LOAEL of 25 mg/kg or 150 mg/kg. Because of the wide dose range used in these studies resulting in a large dose range between the NOAEL and LOAEL values, the NOAEL of 2.5 mg/kg bw derived from these studies may provide a relatively low POD. Taking this consideration into account, the predicted BMDL_10_ values in the present study also seem to be in line with these reported NOAEL values.

## Discussion

The present study aimed to assess the feasibility of using a TEF-coded PBK model for DZN, containing a submodel for its active metabolite DZO, together with reverse dosimetry as an alternative approach to predict rat and human in vivo RBC AChE inhibition dose–response curves for DZN. The models were based on mainly in silico and in vitro data. The rat DZN model was built and evaluated first and used as the basis for developing the human DZN model since less human in vivo data are available for model evaluation (EFSA 2006; JMPR 2016; USEPA 2016). The results obtained reveal that the developed rat model adequately predicted the toxicokinetic profile of DZN in rat, and could also adequately convert the in vitro concentration–response curve to an in vivo dose response curve for DZN-mediated AChE inhibition, resulting in a BMDL_10_ value comparable to the BMDL_10_ reported for inhibition of rat brain AChE and 2.6-fold different from the reported BMDL_10_ for inhibition of rat RBC AChE, respectively (EFSA 2006; JMPR 2016; USEPA 2016). Furthermore, the obtained results also show the predicted BMDL_10_ of human to be 6-fold lower than that of rat, in spite of similar in vitro concentration response curves for DZN or DZO-mediated AChE inhibition. This result indicates that inter-species differences in toxicokinetics of DZN between rat and human play an important role in the ultimate species differences in in vivo toxicity. This 6-fold difference in the BMDL_10_ values as derived for adult populations is smaller than the default uncertainty factor for inter-species differences of 10. However, the actual uncertainty value for inter-species differences may be affected when considering potential species differences in sensitivity at different life stages, with rats showing 2-fold higher sensitivity at younger life stages. On the other hand, when using a QIVIVE based POD derived using human data, use of an inter-species uncertainty factor would no longer be required. In that case an extra uncertainty factor may be considered to account for the fact that the in vitro in-silico QIVIVE approach brings other uncertainties. Altogether, establishment of the actual size of the overall uncertainty factor has to await further studies also including data for potentially vulnerable groups within the human population, like children and pregnant women. Besides, the obtained results also revealed that, in spite of an around 300-fold lower AChE inhibitory potency of DZN than of its active metabolite DZO, DZN still plays a substantial role in the induced AChE inhibition, indicating AChE inhibition induced by DZN should be taken into account in DZN risk assessment. At dose levels higher than 240 mg/kg bw in rat and 50 mg/kg in human, the role of DZN even outweighs that of its active metabolite DZO, mainly due to its substantially higher plasma levels caused by the high dose levels and metabolic capacities for both its bioactivation and detoxification approching saturation. Overall, our findings show that the reverse dosimetry approach combining in vitro data and the TEF-coded PBK models provides a promising tool to predict in vivo dose–response curves for OP-induced AChE inhibition.

The obtained kinetic data revealed that, for both rat and human, CYP450-catalysed conversion of DZN results primarily in detoxification to IMHP rather than giving rise to bioactivation to DZO, which is in line with the conclusion from other studies (Mutch and Williams 2006; Poet et al. 2003; Sams et al. 2004). The *K*_m_ values for these CYP450-mediated conversions appeared to be substantially lower than what was reported before (Poet et al. 2003), a discrepancy most likely due to the concentration ranges used to define the kinetic parameters. In the present study, *K*_m_ values were determined using a range of DZN concentration (1–250 µM) that allowed to fully capture both initial kinetics at low concentrations as well as the saturation phase, while in the literature (Poet et al. 2003), the *K*_m_ was defined based on the activity measured only at concentrations (80–800 µM) exceeding the actual *K*_m_, thus resulting in inaccurate *K*_m_ values. For PON1-mediated detoxification, there were no differences in kinetic parameters for liver and plasma samples of rat or human, indicating that the PON1 activities in these two tissues display similar kinetics, an observation in line with the fact that PON1 in blood originates from synthesis in and release from the liver (Pyati et al. 2015).

Detailed comparison of the species differences between rat and human reveal that rat display a faster metabolic rate for CYP450-mediated bioactivation and detoxification of DZN than human, indicating that at the same exposure level, and assuming similar bioavailability, levels of DZN will be lower in rat than human. This is one of the reasons why differences in kinetics cause a species difference in in vivo toxicity. In addition, PON1-mediated detoxification of DZO to IMHP is faster in rat than human in both liver and blood, counteracting the faster DZO formation from DZN in rat than human. Clearly the PBK models provide a way to evaluate the combined influence of all these differences in kinetics on the ultimate in vivo toxicity. The inter-species kinetic differences in CYP450-mediated conversions may be explained by different CYP450 involved in DZN metabolism in rat and human liver. For rat, metabolism of DZN to DZO and IMHP are mainly catalysed by CYP1A2, CYP2C11, CYP3A2, and CYP2B1/2 (Fabrizi et al. 1999; Ueyama et al. 2007), while in human, the metabolism is mainly mediated by CYP1A1, CYP1A2, CYP2B6, CYP2C19 and CYP3A4 (Ellison et al. 2012; Kappers et al. 2001; Mutch and Williams 2006; Sams et al. 2004). Although the detoxification of DZO in rat and human are both mediated by PON1, in the present study, the rat appeared to be a faster DZO metaboliser than human, which could be explained, at least partly, by higher PON1 activity in rat than human (Berry et al. 2009; Kaliste-Korhonen et al. 1996; Makhaeva et al. 2009). This observation is in line with data reported by Makhaeva et al. (2009) for another OP, indicating an approximately 4-fold faster PON1-mediated hydrolysis of paraoxon hydrolysis by rat than human plasma samples.

To assess the inhibitory capability of DZO on human RBC AChE, the in vitro human AChE inhibition assay was conducted using rhAChE. rhAChE has been widely used to assess AChE inhibition (Amitai et al. 1998; Kaushik et al. 2007; Li et al. 2019; Sultatos 2007), and the IC50 value of DZN obtained using rhAChE in the present study of 14.26 µM is comparable to the value reported previously for natural human RBC AChE (IC50 = 24.45 µM) (Fakhri-Bafghi et al. 2016). Therefore, the use of rhAChE is expected to be adequate to describe the inhibitory profile of DZN and DZO on human RBC AChE. It is also of interest to note that there was no inter-species difference in the in vitro DZO-induced AChE inhibition between rat and human in the present study, and a similar conclusion has been previously reported for dichlorvos, another OP (MacGregor et al. 2005).

In the present study, a TEF-coded PBK model was used to describe free blood concentrations of DZO plus DZN expressed in DZO equivalents at the target site RBC AChE. The results reveal that the free blood concentration of DZN contributes substantially to the DZO equivalents, indicating it is of critical importance to take internal free DZN concentrations into account when conducting DZN risk assessment. The use of the TEF approach is based on the assumptions that (1) DZN and DZO initiate toxicity via the same mode of toxic action (AChE inhibition); (2) their concentration–response curves are parallel; (3) their toxicity is additive (Starr et al. 1999; Watt et al. 2016). The first assumption is supported by the fact that both DZN and DZO induce inhibition of AChE activity. The second assumption was supported by statistical comparison of the hillslope values describing the steepness of the concentration–response curves of DZN and DZO in both human and rat. The comparison showed that the hillslope values of DZN and DZO are similar in both human and rat, with p values of 0.0797 and 0.1504, respectively, indicating the DZN and DZO curves are parallel in both species. In terms of assumption three, the combined effect of DZN and DZO was assessed by incubating rhAChE with DZO only or an equipotent mixture of DZO + DZN in which the concentration of DZN and DZO were selected in such a way that each compound would contribute 50% to the AChE inhibition based on the TEF values of DZN and DZO derived in the present paper. The results (Supplementary data IV) obtained showed that the curves coincided indicating the combined effect of DZN and DZO to be additive. This additive combined effect between DZN and DZO was also observed in the study of Čolović et al. (2011). Different from the conventional TEF approach that defines TEF values based on dose–response curves from in vivo models (USEPA 2016), the TEF values derived in the present study were based on IC50 values derived from in vitro concentration–response curves. This implies that the TEF values do not include the contribution of in vivo toxicokinetics, including absorption, distribution, metabolism, and excretion. These aspects are accounted for by the PBK model itself when performing reverse dosimetry. Using TEF in a toxicokinetic-toxicodynamic (TK-TD) model has been successfully applied in a previous study to predict internal concentrations of a metal mixture and its resulting toxicity (Gao et al. 2016, 2018).

To evaluate whether the currently developed TEF-coded PBK model-facilitated reverse dosimetry approach can be used to determine POD values for DZN risk assessment, BMDL_10_ values obtained from the predicted dose–response curves were compared with EPA reported BMDL_10_ values for both pups and adult rat, showing that the approach provided a reasonable estimation of the BMDL_10_.

Although the currently developed method is promising to be used in future risk assessment, it is of importance to also mention its limitations. The first is that interindividual variations have not (yet) been taken into account. Previously reported studies indicated potentially large interindividual variations in the expression of enzymes involved in DZN metabolism. For the biotransformation of DZN to DZO, this includes an up to 20-fold variation in CYP2C19 human hepatic expression levels, an up to 100-fold variation in CYP2B6 human hepatic expression levels, and an up to 40-fold variation for CYP3A4 expression in liver and small intestine donor tissues (Ellison et al. 2012; Koukouritaki et al. 2004; Lamba et al. 2002; Lang et al. 2001; Tracy et al. 2016; Westlind et al. 1999). Such interindividual variability in CYP450 was reflected by data on DZO formation from DZN showing a 6- to 59-fold difference between 15 human liver samples (Kappers et al. 2001). Similarly, a substantial about 40-fold interindividual human variation in the activity of PON1, the key enzyme for detoxification of DZO, has been observed (Costa et al. 2006). Given that the present study focussed on the average adult population using kinetic data defined with pooled human samples, a further study of the consequences of these interindividual differences for the predicted AChE inhibition remains an interesting topic for future research. The second limitation relates to the reverse dosimetry approach. This PBK-based approach does not account for dynamic changes in AChE activity due to for example AChE regeneration, ageing, degradation, and inhibition. This implies that it can predict acute toxicity but may be less appropriate for prediction of toxicity upon repeated exposure, resulting in inhibition of RBC AChE activity and neurotoxicity of DZN (Hernández et al. 2005). This is further illustrated by the fact the BMDL_10_ values for this subchronic exposure related AChE inhibition are generally lower than the ones reported for inhibition of RBC AChE upon single dose exposure (USEPA 2016). In addition, the potential protective effects of other B-esterases enzymes such as BuChE and CaE were not included in the current study. As reported in previous studies, B-esterases like BuChE and CaE might influence the OP-induced AChE inhibition, by binding the OP so that less oxon-metabolites are available in the circulation to inhibit AChE in vital tissues (Chanda et al. 2002, 1997; Costa 2001, 2006; Jokanović 2009). Such a potential protective role of BuChE and CaE has been investigated especially in animals. Raveh et al. (1997) reported that pretreatment of monkeys with human plasma derived BuChE can protect against toxicity induced by a lethal dose of the OP ethyl-S-(2-diisopropylaminoethyl) methyl-phosphonothiolate. Similarly, a study from Duysen et al. (2011) showed that exposure to 3 mg/kg bw of the OP soman coumarin can be lethal to mice deficient in plasma CaE but not to the wild type mice, indicating a possible potential protective role of CaE. However the role of BuChE and CaE in OP toxicity in human is still not well known (Chanda et al. 2002; Jokanović et al. 2020). The potential protective effect of BuChE and CaE might depend on: (1) the affinity between the enzymes and the respective OP (Chanda et al. 2002); (2) the endogenous level and activity of the enzymes (Chanda et al. 2002; Jokanović et al. 2020); (3) the genotype of the enzymes (Eaton et al. 2008). In the present study this potential protective role of the B-esterases was not specifically considered. This may in theory result in an over-estimation of the predicted dose-dependent AChE inhibition. However, the QIVIVE values in the present study do not seem to overpredict the toxicity as reflected by the good match between the predicted dose–response curves for AChE inhibition in rat and the actual in vivo data available from literature (Fig. 8), suggesting the influence of the B-esterases to be limited if any.

In spite of these limitations the results of the present study show that the DZN TEF-coded PBK model together with QIVIVE appeared a suitable method to predict RBC AChE inhibition upon acute oral exposure to DZN in human and rat. The obtained results indicate an inter-species difference in toxicokinetics of DZN, resulting in the predicted BMDL_10_ of human to be around 6-fold lower than that of rat, indicating that the default uncertainty factor of 10 for inter-species extrapolation might be overprotective. Given the fact that this method is based on mainly an in silico and in vitro approach, it provides an alternative method reducing animal testing for setting PODs in human risk assessment. Furthermore, by replacing relevant parameters (e.g. the absorption and excretion constants) with the data derived based on in silico and in vitro assays, a PBK model for human can be defined and used to derive a POD for human risk assessment without the need for in vivo studies.

## Supplementary Information

Below is the link to the electronic supplementary material.

Supplementary file1 (TIF 644 KB)

Supplementary file2 (TIF 160 KB)

Supplementary file3 (DOCX 352 KB)
