# Supplementary figures and images for: Physiologically based kinetic modelling based prediction of in vivo rat and human acetylcholinesterase (AChE) inhibition upon exposure to diazinon
Source: Arch Toxicol. 2021 Mar 14;95(5):1573–93. doi: 10.1007/s00204-021-03015-1 (PMC8113213; doi:10.1007/s00204-021-03015-1)

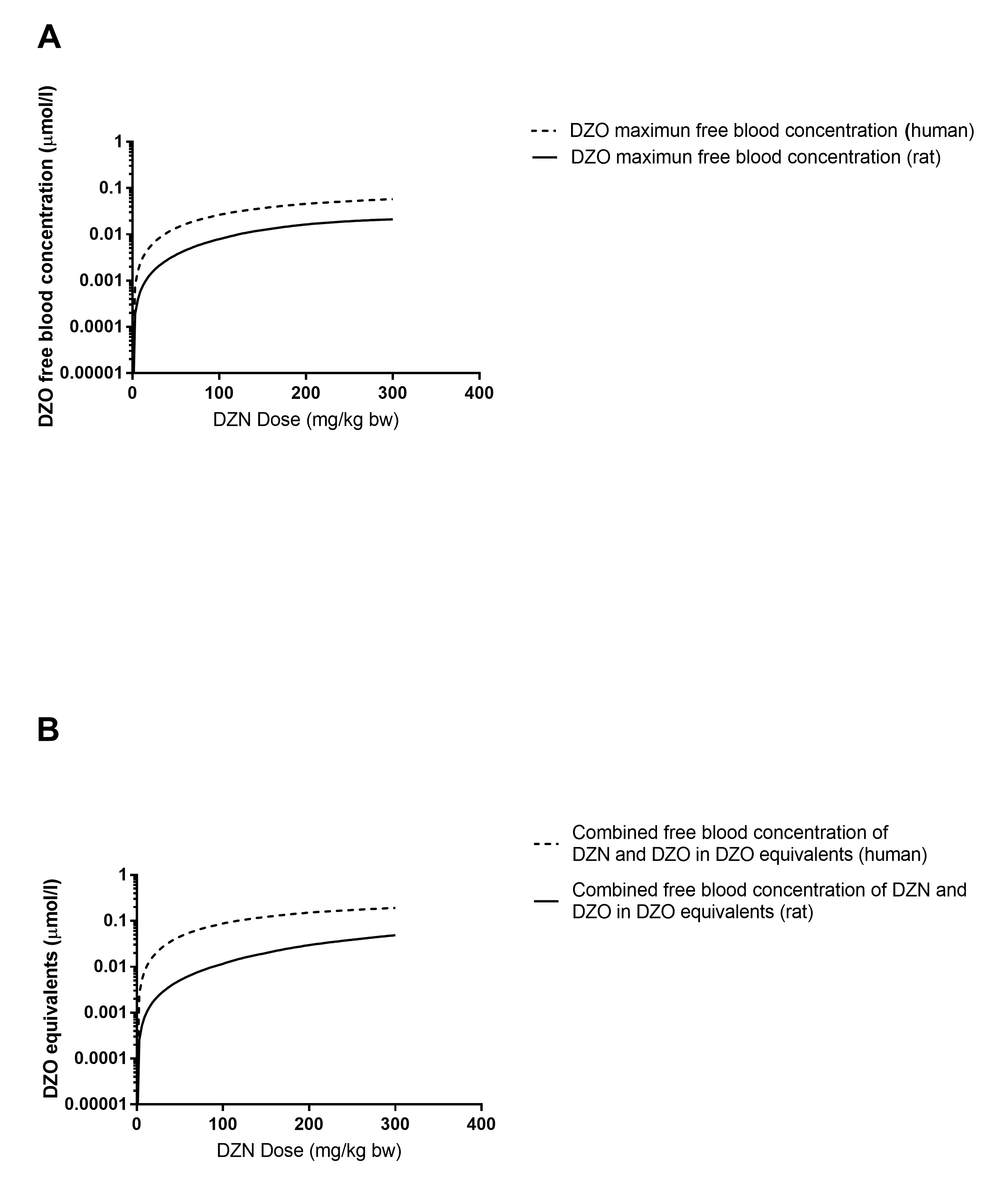

Supplement: Supplementary file 1 — Supplementary file1 (TIF 644 KB) [file 204_2021_3015_MOESM1_ESM.tif]

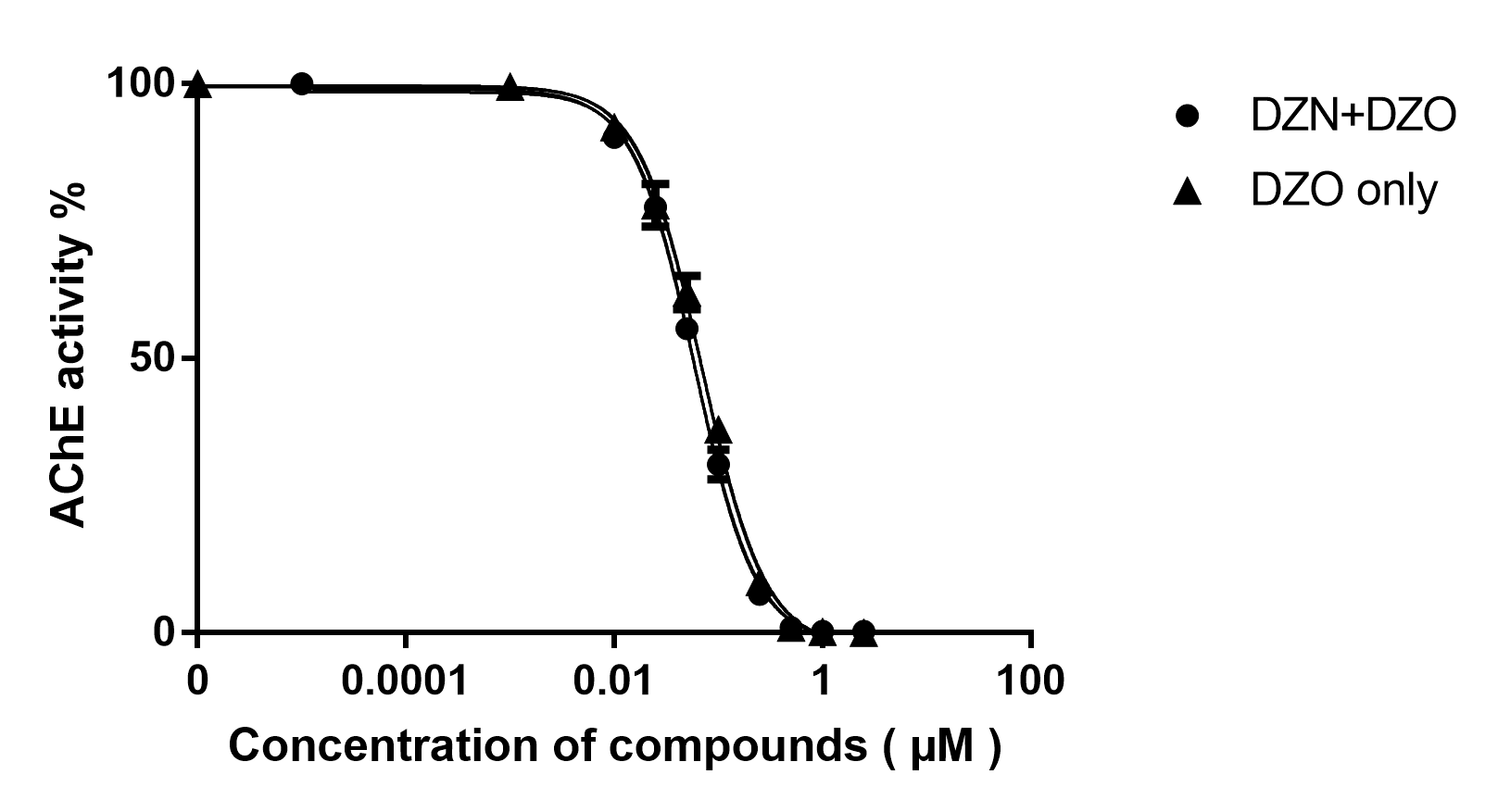

Supplement: Supplementary file 2 — Supplementary file2 (TIF 160 KB) [file 204_2021_3015_MOESM2_ESM.tif]
