# Supplementary material for: Physiologically based kinetic modelling based prediction of in vivo rat and human acetylcholinesterase (AChE) inhibition upon exposure to diazinon
Source: Arch Toxicol. 2021 Mar 14;95(5):1573–93. doi: 10.1007/s00204-021-03015-1 (PMC8113213; doi:10.1007/s00204-021-03015-1)

**Physiologically based kinetic modeling based prediction of in vivo rat and human** **acetylcholinesterase (AChE) inhibition upon exposure to diazinon**

Shensheng Zhao^*,1^, Sebastiaan Wesseling^*^, Bert Spenkelink^*^, Ivonne M.C.M. Rietjens^*^

^*^Division of Toxicology, Wageningen University and Research, Wageningen, The Netherlands

**^1^Corresponding author:**

Shensheng Zhao

Division of Toxicology, Wageningen University and Research

Stippeneng 4, 6708 WE Wageningen,

The Netherlands

E-mail: shensheng.zhao@wur.nl

Supplementary data I

LC-MS/MS acquisition parameters.

| **Compound** | **Precursor**  **ion (m/z)** | **Product**  **ion (m/z)** | **Collision energy (V)** | **Retention time (min)** |
| --- | --- | --- | --- | --- |
| Diazinon (DZN) | 304.80 | 169.15 | -20.0 | 10.49 |
|  |  | 153.15 | -22.0 |  |
|  |  | 97 | -35.0 |  |
| Diazoxon (DZO) | 289.10 | 153.15 | -21.0 | 8.1 |
|  |  | 84.05 | -37.0 |  |
|  |  | 70.05 | -43.0 |  |
| 2-Isopropyl-4-methyl-6-hydroxypyrimidine (IMHP) | 152.70 | 84 | -18.0 | 4.5 |

Supplementary data II

**Differential equations**

**A. Diazinon (DZN) model**

**Needle compartment (IV administration)**

dANeDZN/dt = -kn*ANeDZN

ANeDZN(0) = IV dose

**Stomach compartment (Oral administration)**

dAst1/dt = -KaS*Ast1 -KsI*Ast1

Ast1 (0) = Oral dose * fa

**Intestine compartment (Oral administration)**

dAst2/dt = KsI*Ast1-KaI*Ast2

Ast2 (0) =0

**Liver compartment**

dALDZN / dt = KaS*Ast1 + KaI*Ast2 + QL*(CBDZN - CLDZN/PLDZN) - Vmax1* CLDZN/PLDZN *fuDZN_invivo_/(Km1 + CLDZN/PLDZN*fuDZN_invivo_) - Vmax2* CLDZN/PLDZN*fuDZN_invivo_ /(Km2 + CLDZN/PLDZN*fuDZN_invivo_)

CLDZN = ALDZN/VL

**Fat compartment**

dAFDZN / dt = QF*(CBDZN- CFDZN/PFDZN)

CFDZN= AFDZN/VF

**Richly perfused tissue compartment**

dARDZN / dt = QR*(CBDZN- CRDZN/PRDZN)

CRDZN = ARDZN/VR

**Slowly perfused tissue compartment**

dASDZN / dt = QS*(CBDZN- CSDZN/PSDZN)

CSDZN = ASDZN/VS

**Blood compartment**

dABDZN / dt= kn*ANeDZN +QF* CFDZN/PFDZN + QL* CLDZN/PLDZN + QS* CSDZN/PSDZN + QR* CRDZN/PRDZN - QC*CBDZN

CBCDZN = ABDZN/VB

**B. Diazoxon (DZO) submodel**

**Liver compartment**

dALDZO / dt = QL*(CB DZO - CL DZO /PL DZO) + VMax1* CLDZO/PLDZO*fuDZN_invivo_ /(Km1 + CLDZO/PLDZO*fuDZN_invivo_) - Vmax3* CLDZO/PLDZO*fuDZO_invivo_ /(Km3 + CLDZO/PLDZO*fuDZO_invivo_)

CLDZO = ALDZO/VL

**Fat compartment**

dAFDZO / dt = QF*(CBDZO - CFDZO/PFDZO)

CFDZO = AFDZO/VF

**Richly perfused tissue compartment**

dARDZO / dt = QR*(CBDZO - CRDZO/PRDZO)

CRDZO = ARDZO/VR

**Slowly perfused tissue compartment**

dASDZO / dt = QS*(CBDZO - CSDZO/PSDZO)

CSDZO = ASDZO/VS

**Blood compartment**

dABDZO / dt = QF* CFDZO/PFDZO + QL* CLDZO/PLDZO + QS* CSDZO/PSDZO + QR* CRDZO/PRZO – QC*CBDZO - Vmax4*CBDZO*fuDZO_invivo_ /(Km4 + CBDZO*fuDZO_invivo_)

CBDZO = ABDZO/VB

**C.IMHP compartment**

dAIMHP / dt = (Vmax2* CLDZN/PLDZN *fuDZN_invivo_ /(Km2 + CLDZN/PLDZN*fuDZN_invivo_) + Vmax3* CLDZO/PLDZO*fuDZO_invivo_ /(Km3 + CLDZO/PLDZO*fuDZO_invivo_) + Vmax4*CBDZO*fuDZO_invivo_ /(Km4 + CBDZO*fuDZO_invivo_)) - (KeIMHP*AIMHP)

CBIMHP = AIMHP/VdIMHP

dAIMHPexe / dt = AIMHP*KeIMHP

AIMHPexe (0) =0

**D. DAP compartment**

dADETP / dt = Vmax2* CLDZN/PLDZN*fuDZN_invivo_ /(Km2 + CLDZN/PLDZN*fuDZN_invivo_) - (KeDETP*ADETP)

CBDETP = ADETP/VdDETP

dADEP / dt =(Vmax3* CLDZO/PLDZO*fuDZO_invivo_ /(Km3 + CLDZO/PLDZO*fuDZO_invivo_) + Vmax4*CBDZO*fuDZO_invivo_ /(Km4 + CBDZO*fuDZO_invivo_)) – (KeDEP*ADEP)

CBDEP=ADEP/VdDEP

dADAP / dt = (Vmax2* CLDZN/PLDZN *fuDZN_invivo_ /(Km2 + CLDZN/PLDZN*fuDZN_invivo_) + Vmax3* CLDZO/PLDZO*fuDZO_invivo_ /(Km3 + CLDZO/PLDZO*fuDZO_invivo_) + Vmax4*CBDZO*fuDZO_invivo_ /(Km4 + CBDZO*fuDZO_invivo_)) - (KeDETP*ADETP) – (KeDEP*ADEP)

CBDAP = ADAP/VdDAP

dADAPexe / dt= (KeDETP*ADETP) + (KeDEP*ADEP)

ADAPexe (0) =0

DZOTEQ=CBDZNPP*TEFDZN+CBDZOPP*TEFDZO

| Abbreviation | Variable | Unit |
| --- | --- | --- |
| DZN | Diazinon |  |
| DZO | Diazoxon |  |
| IMHP | 2-isopropyl-4-methyl-6-hydroxypyrimidine |  |
| DETP | diethylthiophosphate |  |
| DAP | Dialkylphosphate (sum of DEP and DETP in present study) |  |
| DEP | diethylphosphate |  |
| VF | Volume of fat tissue | L or Kg |
| VL | Volume of liver tissue | L or Kg |
| VB | Volume of blood | L or Kg |
| VR | Volume of richly perfused tissue | L or Kg |
| VS | Volume of slowly perfused tissue | L or Kg |
| QC | Cardiac output | L/hr |
| QF | Blood flow to fat tissue | L/h |
| QL | Blood flow to liver tissue | L/h |
| QS | Blood flow to slowly perfused tissue | L/h |
| QR | Blood flow to richly perfused tissue | L/h |
| PFDZN | Fat/blood partition coefficient of DZN |  |
| PLDZN | Liver/blood partition coefficient of DZN |  |
| PRDZN | Richly perfused tissue/blood partition coefficient of DZN |  |
| PSDZN | Slowly perfused tissue/blood partition coefficient of DZN |  |
| PFDZO | Fat/blood partition coefficient of DZO |  |
| PLDZO | Liver/blood partition coefficient of DZO |  |
| PRDZO | Richly perfused tissue/blood partition coefficient of DZO |  |
| PSDZO | Slowly perfused tissue/blood partition coefficient of DZO |  |
| ANeDZN | Amount of DZN in needle | µmol |
| kn | The transport rate of DZN from needle to blood | /h |
| IV dose | Amount of DZN is administered via Intravenous way | mg/kg bw |
| Oral dose | Amount of DZN is administered via oral | mg/kg bw |
| Ast1 | Amount of DZN remaining in Stomach | µmol |
| Ast2 | Amount of DZN remaining in Intestine | µmol |
| KaS | First order rate constant for absorption DZN from stomach | /h |
| KaI | First order rate constant for absorption DZN from intestine | /h |
| KsI | First order rate constant for transfer of DZN from stomach to intestine | /h |
| fa | Fraction of absorption | % |
| KeIMHP | First order rate constant for elimination of IMHP from body | /h |
| KeDETP | First order rate constant for elimination of DETP from body | /h |
| KeDEP | First order rate constant for elimination of DEP from body | /h |
| Vd IMHP | IMHP volume of distribution | L |
| VdDETP | DETP volume of distribution | L |
| VdDEP | DEP volume of distribution | L |
| Vmax1 | Maximum rate for metabolism from DZN to DZO | µmol/h |
| Km1 | Michaelis-Menten constant for metabolism from DZN to DZO | µM |
| Vmax2 | Maximum rate for metabolism from DZN to IMHP/DETP (liver) | µmol/h |
| Km2 | Michaelis-Menten constant for metabolism from DZN to IMHP/DETP (liver) | µM |
| Vmax3 | Maximum rate for metabolism from DZO to IMHP/DEP (liver) | µmol/h |
| Km3 | Michaelis-Menten constant for metabolism from DZO to IMHP/DEP (liver) | µM |
| Vmax4 | Maximum rate for metabolism from DZO to IMHP/DEP (Blood) | µmol/h |
| Km4 | Michaelis-Menten constant for metabolism from DZO to IMHP/ DEP(Blood) | µM |
| ALDZN | Amount of DZN in the liver | µmol |
| CVLDZN | DZN concentration in venous blood leaving the liver | µmol/L |
| CLDZN | DZN concentration in the liver | µmol/L |
| AFDZN | Amount of DZN in the fat tissue | µmol |
| CVFDZN | DZN concentration in venous blood leaving the fat tissue | µmol/L |
| CFDZN | DZN concentration in the fat tissue | µmol/L |
| ARDZN | Amount of DZN in the richly perfused tissue | µmol |
| CVRDZN | DZN concentration in venous blood leaving the richly perfused tissue | µmol/L |
| CRDZN | DZN concentration in the richly perfused tissue | µmol/L |
| ASDZN | Amount of DZN in the slowly perfused tissue | µmol |
| CVSDZN | DZN concentration in venous blood leaving the slowly perfused tissue | µmol/L |
| CSDZN | DZN concentration in the slowly perfused tissue | µmol/L |
| ABDZN | Amount of DZN in blood | µmol |
| CBDZN | DZN concentration in arterial blood entering each compartment | µmol/L |
| ALDZO | Amount of DZO in liver tissue | µmol |
| CBDZO | DZO concentration in arterial blood entering each compartment | µmol/L |
| CVLDZO | DZO concentration in venous blood leaving the liver | µmol/L |
| CLDZO | DZO concentration in the liver | µmol/L |
| AFDZO | Amount of DZO in fat tissue | µmol |
| CVFDZO | DZO concentration in venous blood leaving the fat tissue | µmol/L |
| CFDZO | DZO concentration in the fat tissue | µmol/L |
| ARDZO | Amount of DZO in the richly perfused tissue | µmol |
| CVRDZO | DZO concentration in venous blood leaving the richly perfused tissue | µmol/L |
| CRDZO | DZO concentration in the richly perfused tissue | µmol/L |
| ASDZO | Amount of DZO in the slowly perfused tissue | µmol |
| CVSDZO | DZO concentration in venous blood leaving the slowly perfused tissue | µmol/L |
| CSDZO | DZO concentration in the slowly perfused tissue | µmol/L |
| ABDZO | Amount of DZO in blood | µmol |
| AIMHP | Amount of IMHP in body | µmol |
| AIMHPexe | Amount of IMHP eliminate to urine | µmol |
| ADETP | Amount of DETP in body | µmol |
| ADEP | Amount of DEP in body | µmol |
| ADAPexe | Amount of DAP eliminate to urine | µmol |
| DZOTEQ | Free blood TEF equivalent DZO concentration | µmol/L |
| CBDZNPP | Free blood DZN concentration | µmol/L |
| CBDZOPP | Free blood DZO concentration | µmol/L |
| TEFDZN | Toxic equivalency factor of DZN |  |
| TEFDZO | Toxic equivalency factor of DZO |  |
| fuDZN_in vivo_ | Free fraction of DZN in vivo |  |
| fuDZO_in vivo_ | Free fraction of DZO in vivo |  |

Supplementary data III

The PBK model based predicted (A) DZN dose-dependent maximum free blood concentration of DZO and (B) DZN dose-dependent combined free blood concentration of DZN plus DZO expressed in DZO equivalents in rat (solid line) and human (dash line)


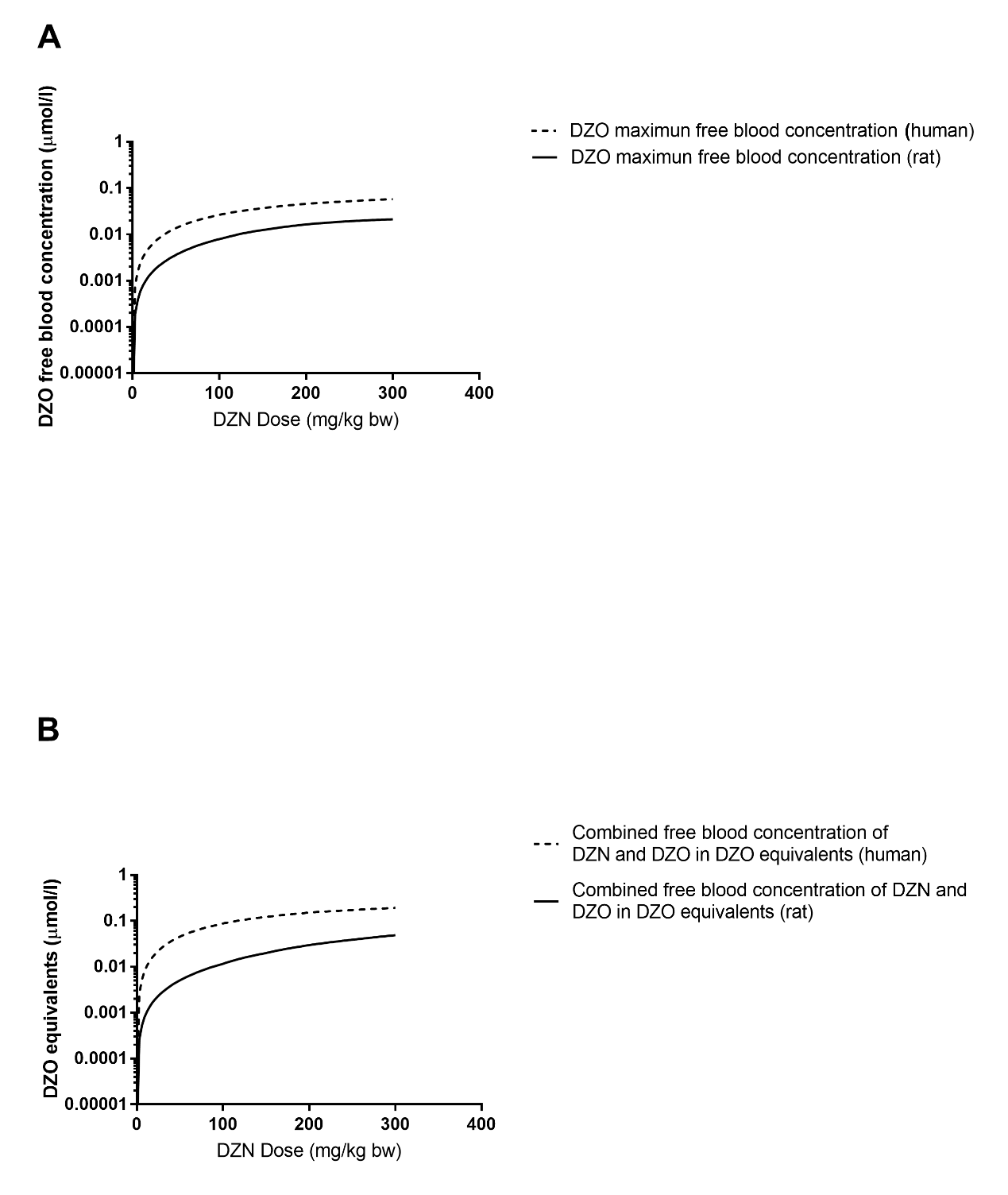


Supplementary data IV

Effect of increasing concentration of DZO only (triangles) and an equipotent mixture of DZO+DZN (circles) on acetylcholinesterase (AChE) activity of human at 37℃. Each value represents the mean ± SD of two independent experiments.


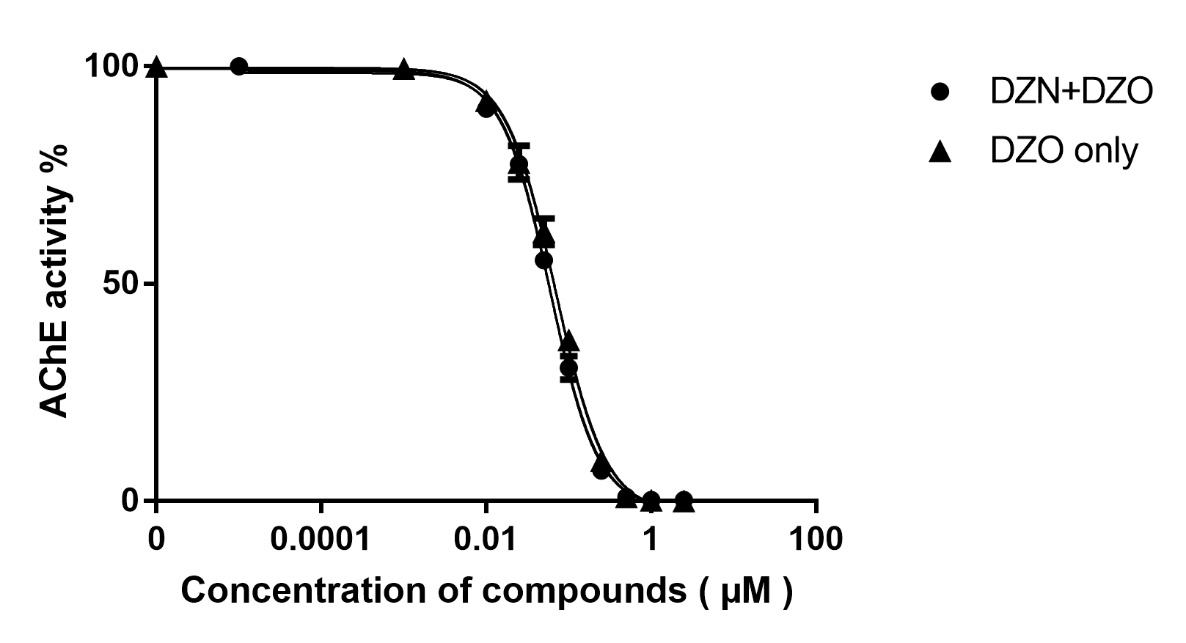

Supplement: Supplementary file 3 — Supplementary file3 (DOCX 352 KB) [file 204_2021_3015_MOESM3_ESM.docx]
